# Cell cycle controls long-range calcium signaling in the regenerating epidermis

**DOI:** 10.1083/jcb.202302095

**Published:** 2023-04-27

**Authors:** Jessica L. Moore, Dhananjay Bhaskar, Feng Gao, Catherine Matte-Martone, Shuangshuang Du, Elizabeth Lathrop, Smirthy Ganesan, Lin Shao, Rachael Norris, Nil Campamà Sanz, Karl Annusver, Maria Kasper, Andy Cox, Caroline Hendry, Bastian Rieck, Smita Krishnaswamy, Valentina Greco

**Affiliations:** 1 Department of Genetics, Yale University School of Medicine, New Haven, CT, USA; 2 Department of Neuroscience, Yale University School of Medicine, New Haven, CT, USA; 3 Department of Cell Biology, UConn Health, Farmington, CT, USA; 4 Department of Cell and Molecular Biology (CMB), Karolinska Institutet, Stockholm, Sweden; 5 Helmholtz Pioneer Campus, Helmholtz Munich, Neuherberg, Germany; 6 Department of Computer Science, Yale University, New Haven, CT, USA; 7 Applied Mathematics Program, Yale University, New Haven, CT, USA; 8 Program for Computational Biology and Bioinformatics, Yale University, New Haven, CT, USA; 9 Wu Tsai Institute, Yale University, New Haven, CT, USA; 10 Department of Cell Biology, Yale University School of Medicine, New Haven, CT, USA; 11 Department of Dermatology, Yale University School of Medicine, New Haven, CT, USA; 12 Yale Stem Cell Center, Yale University School of Medicine, New Haven, CT, USA; 13 Yale Cancer Center, Yale University School of Medicine, New Haven, CT, USA

## Abstract

Skin homeostasis is maintained by stem cells, which must communicate to balance their regenerative behaviors. Yet, how adult stem cells signal across regenerative tissue remains unknown due to challenges in studying signaling dynamics in live mice. We combined live imaging in the mouse basal stem cell layer with machine learning tools to analyze patterns of Ca^2+^ signaling. We show that basal cells display dynamic intercellular Ca^2+^ signaling among local neighborhoods. We find that these Ca^2+^ signals are coordinated across thousands of cells and that this coordination is an emergent property of the stem cell layer. We demonstrate that G2 cells are required to initiate normal levels of Ca^2+^ signaling, while connexin43 connects basal cells to orchestrate tissue-wide coordination of Ca^2+^ signaling. Lastly, we find that Ca^2+^ signaling drives cell cycle progression, revealing a communication feedback loop. This work provides resolution into how stem cells at different cell cycle stages coordinate tissue-wide signaling during epidermal regeneration.

## Teaser

Moore, Bhaskar, Gao, et al. combine live imaging of the mouse epidermis and machine learning to study the role and regulation of calcium signaling within the stem cell layer. They find that cells in G2 are necessary for coordinated tissue-wide communication and reveal a feedback loop between cell cycle and calcium signaling.

## Introduction

Each day our bodies make and lose billions of cells (Sender and Milo, 2021). This regenerative capacity is based on the ability to orchestrate fate decisions within an actively cycling stem cell pool, resulting in a balanced production of new cells (by division) and loss of cells (by differentiation or apoptosis). In epithelial regeneration across a number of organisms, these stem cell behaviors are directly coupled within local neighborhoods (Mesa et al., 2018; Liang et al., 2017). Yet, how stem cells communicate with their neighbors at various distances remains largely unexplored due to the complexity of capturing signaling dynamics across space and time in a live, uninjured setting.

In response to injury and during development, epithelial stem cells are known to coordinate their regenerative behaviors via Ca^2+^ signaling (Restrepo and Basler, 2016; Balaji et al., 2017; Ohno and Otaki, 2015; Celli et al., 2021). Ca^2+^ signaling is implicated in a diversity of cellular functions and has become recognized as critical to stem cell function across systems (Snoeck, 2020; Rosendo-Pineda et al., 2020). An abundance of in vitro studies have established that the temporal dynamics of Ca^2+^ signaling are tightly regulated and can differentially encode function, allowing for its versatility as a signaling pathway (Dolmetsch et al., 1997; Dolmetsch et al., 1998; De Koninck and Schulman, 1998). Much of the work to understand the function and dynamics of Ca^2+^ signaling in vivo has come from the neurobiology field, where it is possible to measure Ca^2+^ signaling across broad spatial and temporal scales; however, the live in vivo dynamics of Ca^2+^ signaling in other tissue contexts remains relatively unexplored. Specifically, the in vivo spatiotemporal characteristics of Ca^2+^ signaling during tissue regeneration and the fundamental mechanisms regulating these dynamics are unclear.

Here, we explore how stem cells communicate with one another and their neighbors in a regenerative context, in vivo, focusing on the basal stem cell layer of the mouse epidermis. The basal layer is heterogeneous and comprised of stem cells and differentiation-committed neighbors in various cell cycle stages. Recent work has provided evidence of local coordination of regenerative behaviors, where cell fates are not only influenced by their direct neighbors (Mesa et al., 2018) but also by large-scale organization, where dynamic behaviors of diverse cell types are coordinated (Hiratsuka et al., 2015; Park et al., 2021). It remains unclear how coordinated regenerative behavior is communicated across epidermal tissue. While upregulation of intracellular Ca^2+^ levels is essential for the terminal steps of epidermal differentiation in the skin (Hennings et al., 1980; Yuspa et al., 1989; Elias et al., 2002; Koizumi et al., 2004; Behne et al., 2011; Celli et al., 2011, 2012; Darbellay et al., 2014; Kumamoto et al., 2017; Matsui et al., 2021), the role of Ca^2+^ signaling dynamics and its regulation have not been explored in the basal stem cell layer.

**Figure 1.**
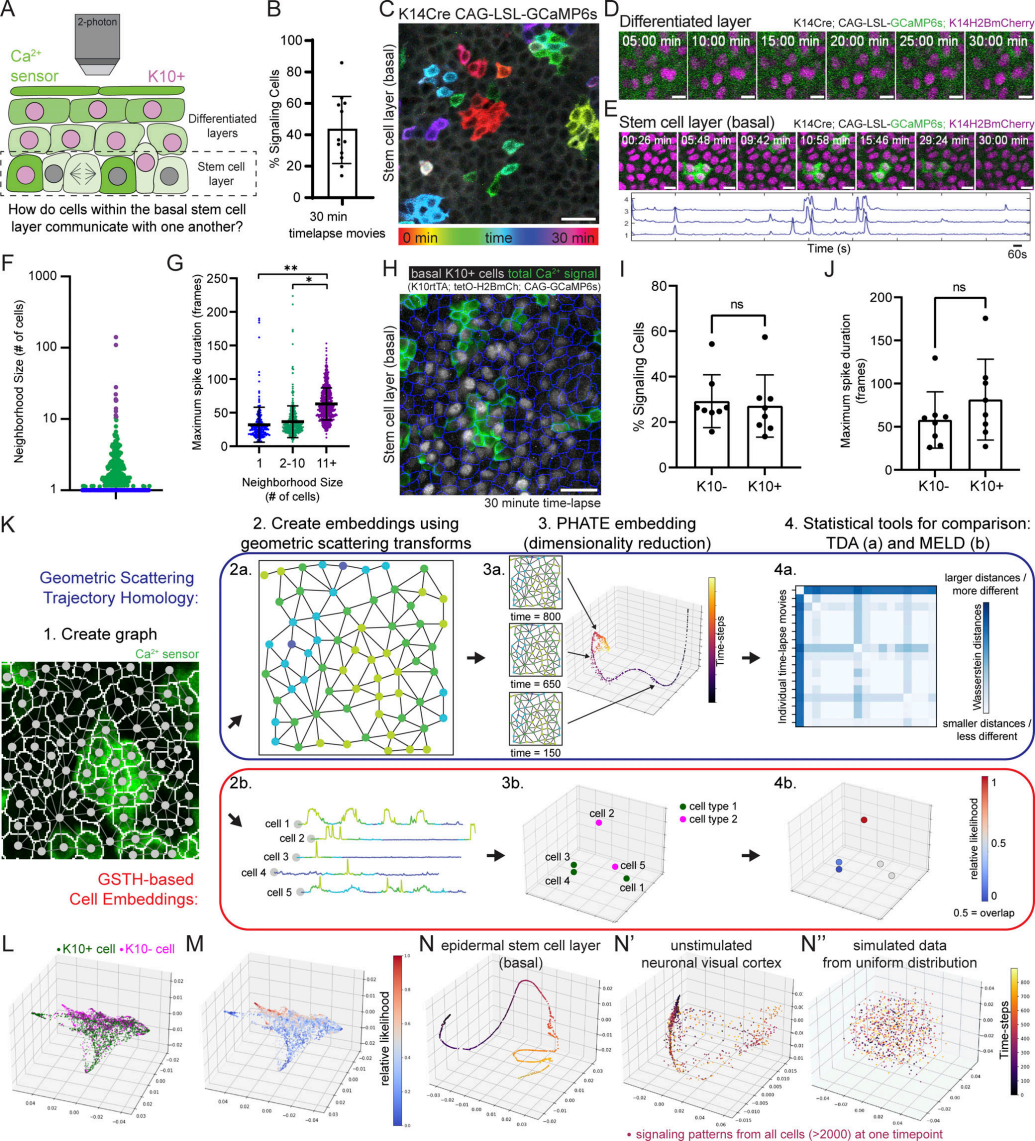
**The epidermal stem cell layer cohesively carries out coordinated Ca**^**2+**^ **signaling at long range. (A)** Schematic of intravital imaging of Ca^2+^-sensor mice. **(B)** Percent of epidermal basal cells spiking at least once during 30-min recording of Ca^2+^ signaling in a live mouse. *n* = 12 movies from six mice. **(C)** Max intensity projection of GCaMP6s signal from 30-min recording of the stem cell layer, showing a diversity of signaling patterns. Color scale indicates GCaMP6s signal across time. Scale bar: 25 µm. **(D)** Lack of Ca^2+^ signaling in the differentiated spinous layer over 30 min. Scale bars: 10 µm. **(E)** Region of the stem cell layer where a cluster of three cells spikes repeatedly over 30 min. Normalized fluorescence intensity plotted across time for each spiking cell is below. Scale bars: 10 µm. **(F)** Neighborhood size of cells with spatiotemporally localized Ca^2+^ signaling from 30-min recording of the epidermal basal stem cell layer. Purple, blue, and green dots represent the three spatial patterns. *n* = 6 recordings from three mice. **(G)** Maximal spike duration (maximal number of 2-s frames between the start and end of individual Ca^2+^ events) for three spatial patterns of Ca^2+^ signaling. *P = 0.0213, **P = 0.0056, nested one-way ANOVA, *n* = 6 recordings. **(H)** Max intensity projection of 30-min recording of the stem cell layer of Ca^2+^-sensor mouse with a K10 reporter. Nuclear signal in grayscale reports K10 expression and differentiation commitment. Cell segmentation is in blue and any Ca^2+^ activity from the 30 min is in green. Scale bar: 25 µm. **(I)** Percent of basal K10-reporter-positive and K10-reporter-negative cells spiking at least once during 30 min of Ca^2+^ signaling in a live mouse. ns = P *>* 0.05, unpaired two-tailed Student’s *t* test, *n* = 8 movies from five mice. **(J)** Maximal spike duration of Ca^2+^ transients in K10-reporter-positive versus K10-reporter-negative basal cells. ns = P *>* 0.05, unpaired two-tailed Student’s *t* test, *n* = 8 movies from five mice. **(K)** GSTH (a in blue) and GSTH-based Cell Embeddings (b in red) workflows. Step 1: The cellular graph is created based on spatial adjacency (nodes shown on a segmented image of stem cell layer Ca^2+^ signaling). Step 2: Timepoint (a) and cell (b) embeddings are created using the geometric scattering transform. Step 3: PHATE (dimensionality reduction method) visualization of time trajectory (a) or cell embeddings (b). Step 4: Statistical tools for comparison. Above: Topological data analysis—persistence diagrams and Betti curves describing the geometry of the trajectories and comparisons via Wasserstein distances. Below: Overlap of two populations is computed using MELD. **(L)** Representative PHATE plot of cell embeddings from K10 experiment in H. Each dot represents a single cell; its position in space represents similarity of its Ca^2+^ signaling to other cells’ signaling; differentiation-committed K10-reporter-positive cells are shown in green and K10-reporter-negative stem cells are shown in magenta. **(M)** MELD comparison of K10-reporter-positive versus K10-reporter-negative signaling patterns. Values close to 0.5 represent the most overlap between the two populations. **(N–N”)** Representative PHATE visualization of coordinated Ca^2+^ signaling in the homeostatic epithelial stem cell layer (N) versus disorganized Ca^2+^ signaling in the quiescent neuronal visual cortex (N’) and simulated data using a uniform distribution of values (N”).

To address this, we evolved our two-photon microscopy system (Pineda et al., 2015; Rompolas et al., 2016) to capture higher-resolution images in Ca^2+^-sensor mice (Chen et al., 2013), allowing us to simultaneously capture dynamic Ca^2+^ signaling at the single cell level, in a tissue-wide manner (i.e., across thousands of basal cells; Fig. 1 A). We observed the local spread of Ca^2+^ signals across neighborhoods of up to 10 basal cells throughout the basal layer. To better understand Ca^2+^ signaling dynamics over time, we developed an unsupervised data-driven computational analysis framework—Geometric Scattering Trajectory Homology (GSTH)—to analyze and model signaling dynamics both locally between neighboring cells and at a tissue-wide level. Using this approach together with mouse models, we show that Ca^2+^ signaling is coordinated at large distances across thousands of cells. We find that cells in the G2 phase of the cell cycle are necessary for normal levels and patterns of Ca^2+^ signaling. Further, we demonstrate that most other cells in the basal layer (G1 cells) have high junctional Cx43 levels that allow Ca^2+^ signals to be coordinated globally, across neighborhoods of cells. Finally, we interrogate the function of coordinated Ca^2+^ signaling within the stem cell layer, finding that it promotes cell cycle progression, revealing a feedback loop between cell cycle and Ca^2+^ signaling. Together, our results provide insight into the relationship between the cell cycle in the stem cell pool and Ca^2+^ signaling, and how coordinated Ca^2+^ signaling helps maintain tissue homeostasis.

## Results

### Epidermal stem cell layer displays local neighborhoods of Ca^2+^ signaling

Under homeostatic conditions, epidermal basal cells either progress through the cell cycle toward division or exit into the suprabasal differentiated layer (Fig. 1 A). Proper coordination of these behaviors is necessary for healthy skin regeneration and requires communication among basal cells. To understand the characteristics and regulation of communication in the basal stem cell compartment, we turned to Ca^2+^ signaling, which is essential for regenerative behaviors, such as proliferation, in other epithelial contexts (Restrepo and Basler, 2016; Balaji et al., 2017; Ohno and Otaki, 2015; Celli et al., 2021). We generated mice with a Ca^2+^ sensor expressed in all epidermal cells (*K14-Cre; Rosa26-CAG-LSL-GCaMP6s*) and combined this with a nuclear marker (*K14-H2B-mCherry*). Live imaging of the basal stem cell layer (Pineda et al., 2015; Rompolas et al., 2016) revealed highly variable levels of Ca^2+^ activity within the basal cells, with 43.1 ± 21.4% of cells showing at least one Ca^2+^ transient in a given 30-min time frame (Video 1 and Fig. 1 B).

**Video 1.** **Long-range coordination of clusters of Ca**^**2+**^ **signaling across the basal stem cell layer.** Intravital imaging of the epithelial stem cell layer of Ca^2+^ sensor mice (*K14-Cre; R26-CAG-LSL-GCaMP6s; K14-H2B-mCherry*). Magenta marks the nuclei of each basal cell and green fluorescence intensity represents relative cytosolic Ca^2+^ levels. Transverse views of the infundibulum of hair follicles marked with HF. Frames were taken every 2 s for a total duration of 30 min. Movie shows dynamics at 40 frames per second or 80× the actual timescale. Scale bar: 25 μm.

To understand how cells orchestrate Ca^2+^ dynamics within the stem cell compartment, we then imaged large epidermal regions every 2 s for 30 min and temporally color-coded each frame of the recording to simultaneously visualize all the Ca^2+^ signaling patterns (Fig. 1 C). We observed distinct spatiotemporal patterns of Ca^2+^ signaling within the stem cell layer: in some cases, single cells spiked quickly in isolation, whereas in others, neighborhoods of cells spiked simultaneously or in a propagating wave. The dynamic nature of these intercellular signaling events was a feature of the epidermal stem cell layer, while the differentiated and quiescent suprabasal (spinous) layer directly above showed no signaling activity (Fig. 1 D).

**Figure S1.**
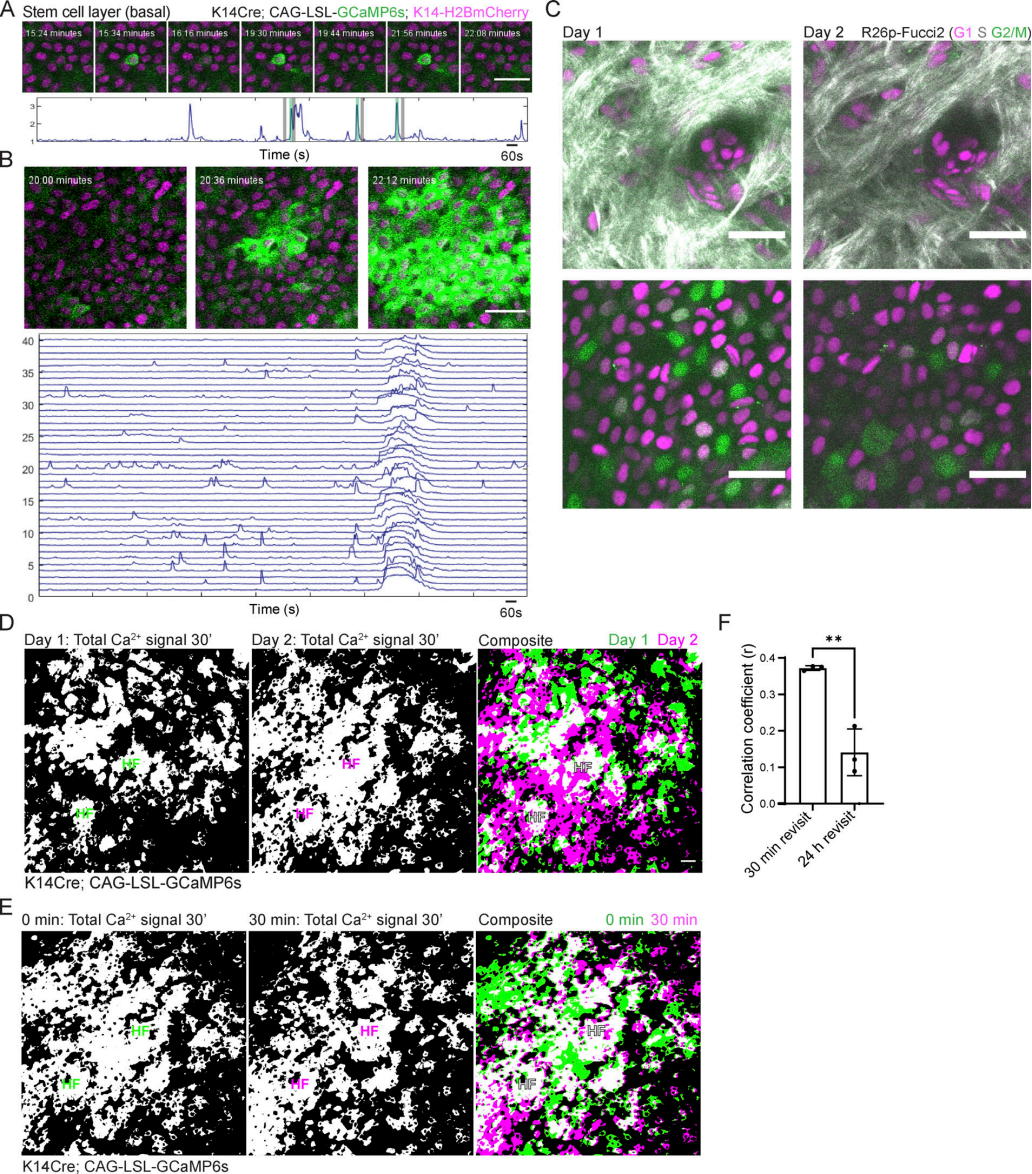
**Pervasive, fast Ca**^**2+**^ **dynamics are specific to the regenerative basal layer of the epidermis. (A)** Region of the basal stem cell layer where a single cell spikes repeatedly over 30 min of imaging (*K14-Cre; Rosa26-CAG-LSL-GCaMP6s; K14-H2B-mCherry*). Normalized fluorescence intensity plotted over the duration of 30-min timelapse is below. Black and green bars indicate timepoints corresponding to the snapshots above. Scale bars: 25 µm. **(B)** Different regions of the stem cell layer, where a large group of cells exhibit an ICW. Normalized fluorescence intensity plotted over the 30-min timelapse for 40 of the cells involved in the ICW is below. Scale bars: 25 µm. **(C)** Revisit of the same region of a *Rosa26p-Fucci2* mouse at 0 and 24 h. Top panel shows dermis and bottom panel shows the epidermal basal layer. Collagen is in white, G1 cells are in magenta, S cells are double positive for magenta and green and shown in gray, and G2 and M cells are in green. Scale bars: 25 µm. **(D)** Maximum intensity projection of all optical sections of 30-min timelapses from the same region of the epidermis at 0 min and 24 h. To the right, a composite image of the max intensity projections at 0 (green) and 24 h (magenta). White indicates overlapping regions of Ca^2+^ signaling. Scale bars: 25 µm. Transverse views of the top of the infundibulum region of hair follicles marked with HF to orient us in revisiting the region. **(E)** Maximum intensity projection of all optical sections of a 30-min timelapse at 0 and 30 min of the same region of the epidermis. To the right, composite image of the same region at 0 (green) and 30 min (magenta), where white indicates overlapping regions of Ca^2+^ activity. Transverse views of the top of the infundibulum region of hair follicles marked with HF to orient us in revisiting the region. **(F)** Correlation coefficient quantification of pixel overlap of Ca^2+^ signaling during 30-min timelapses from revisits of the basal layer taken at 30 min and 24 h timepoints. **P *<* 0.01, unpaired two-tailed Student’s *t* test. *n* = 3 mice.

To systematically quantify Ca^2+^ transients from each individual cell in the stem cell layer and to understand the relationship in time and space between these Ca^2+^ transients, we adapted existing tools (Romano et al., 2017; Giovannucci et al., 2019) to segment individual cells, identify peaks of increased intracellular Ca^2+^, and define a simple graph of connected Ca^2+^ signaling (nodes were connected if the cells they represented were direct neighbors and spiked within 10 s of their neighbor). We then quantified the number of connected nodes (cells) in each clustered signaling neighborhood and explored the temporal dynamics for each neighborhood size. Using this approach, we found that most events within the stem cell layer were either single cells that spiked in isolation from their neighboring cells (65.88 ± 2.65%; Fig. S1 A) or spatiotemporally clustered transients across two or more neighboring cells (31.27 ± 1.96%; Fig. 1, E and F). We also observed rare Ca^2+^-signaling waves that spread across hundreds of cells (2.85 ± 1.00%; Fig. S1 B). We next quantified the duration of the longest Ca^2+^ transient per cell, which we termed maximal spike duration. We found that Ca^2+^ transients in the largest neighborhoods of cells persist longer than in single cells or small neighborhoods (Fig. 1 G). These data show that the stem cell layer is characterized by the local spread of Ca^2+^ signaling across neighborhoods of mostly 1–10 cells with short temporal characteristics.

To determine how much of the basal layer participates in Ca^2+^ signaling, we asked whether regions in the basal stem cell layer with high levels of homeostatic Ca^2+^ signaling are temporary or persistent. To this end, we recorded Ca^2+^ signaling in a large 500 µm × 500 µm region (encompassing about 2,500 epidermal basal cells) for 30 min and revisited the same region 24 h later. During a 24-h period, many cells in the basal layer progress through different cell cycle phases, and this population of cells is dynamic (Fig. S1 C). We quantified the correlation between the total GCaMP6s Ca^2+^ reporter signal in the basal layer at 0 and 24 h. Image correlation coefficients closer to 1 represented a high overlap of signal, and correlation coefficients of 0 meant that only 50% of the signal overlapped between the two timepoints. A comparison of active Ca^2+^ signaling across the 24-h period revealed that Ca^2+^ signaling is not spatially persistent but rather changes regionally with time, encompassing most of the stem cell layer. The correlation of signaling activity between 24 h (0.1411 ± 0.06416) was significantly lower than the signaling correlation of a region at 0 and 30 min (0.3723 ± 0.0064; Fig. S1, D–F). Together, these results demonstrate that Ca^2+^ signaling is dynamic and not restricted to certain hotspot domains of the basal stem cell layer.

Within the stem cell layer of the epidermis, a subset of cells undergoes a gradual, multiday differentiation process as they prepare to exit this layer (Cockburn et al., 2022). We wondered whether Ca^2+^ signaling was similar between these differentiation-committed cells and their stem cell neighbors. This subset of basal cells (about 40%) expresses the early differentiation marker keratin 10 (K10; Schweizer et al., 1984; Braun et al., 2003), allowing us to take advantage of a live reporter system in which the *Krt10* promoter drives H2B-mCherry fluorescence and follow Ca^2+^ dynamics in differentiation-committed basal cells (*Rosa26-CAG-GCaMP6s; K10-rtTA*; *Col1a1-tetO-H2B-mCherry*; Muroyama and Lechler, 2017; Cockburn et al., 2022). This live reporter captures the dynamics of most cells that have committed to differentiate with about 80% of cells with K10 protein expression marked by the *Krt10*-based reporter transgene (Cockburn et al., 2022). After imaging large epidermal regions every 2 s for 30 min, K10-reporter-positive and -negative cells showed similar participation in Ca^2+^ signaling and length of Ca^2+^ spikes (Fig. 1, H–J). These results demonstrate that differentiation-committed progenitors and their stem cell neighbors display similar Ca^2+^ dynamics in some spatiotemporal dimensions, such as participation in Ca^2+^ signaling and spike duration.

### GSTH

Understanding in vivo signaling dynamics of any molecular pathway at a large scale is a formidable challenge, especially in highly dynamic regenerative tissues due to both the spatiotemporal heterogeneity of signaling dynamics as well as the inherent complexity of the tissue (i.e., thousands of cells, heterogeneous states of the signaling cells, dynamic cell behaviors, etc.). To analyze signaling dynamics in our system, we developed an unsupervised machine learning method called Geometric Scattering Trajectory Homology (GSTH), which captures spatial and temporal signaling patterns in a highly applicable manner and allows us to compare the Ca^2+^ signaling behavior of cells under many conditions across multiple scales (Bhaskar et al., 2023 *Preprint*).

GSTH produces 3D plots, which show a low-dimensional representation of the scattering transform, i.e., the concatenated zeroth-, first-, and second-order scattering coefficients, computed using the raw signaling data (Fig. 1 K). This representation is computed using PHATE (Moon et al., 2019), a dimensionality reduction and manifold visualization technique that preserves both local and global nonlinear structures. We use PHATE plots to visualize and analyze the data in two distinct ways: firstly, to quantify temporal changes in Ca^2+^ signaling using GSTH time embeddings, and secondly, to understand cell-specific heterogeneity in Ca^2+^ signaling patterns. We elaborate on the intuitiveness and interpretability of the PHATE plots for both use cases below.

#### GSTH—Temporal dynamics in Ca^2+^ signaling (Fig. 1 K, top)

To quantify temporal dynamics, we compute scattering coefficients for each timepoint by aggregating over cells in the scattering transform (Fig. 1 K, step 2a). We visualize the resulting GSTH timepoint embedding in 3D using PHATE, where each point in the PHATE plot corresponds to a single timepoint in the input data (Fig. 1 K, step 3a). Two points are close together if the underlying spatial signaling pattern across all cells at the corresponding timepoints is similar. For example, consider a tissue comprised of cells oscillating synchronously. The PHATE plot in this example will appear as a loop-like trajectory, with the size of the loop proportional to the period of oscillation.

Next, we use persistent homology to quantify the shape of the trajectory in the PHATE plots. Dimension 0 homology (denoted *H0*) measures the connectivity between the points, whereas dimension 1 homology (denoted *H1*) tracks the presence of loops in the trajectory. *H0* distinguishes between “continuous” and “discontinuous” (large gaps between successive timepoints) trajectories. Discontinuous trajectories arise in datasets with spontaneous, “bursty,” or uncorrelated signaling activity. A persistent topological loop (*H1*) is created when a section of the trajectory takes the shape of a closed curve, with two nonconsecutive timepoints close to each other in the PHATE plot. These loops indicate the presence of quasi-periodic activity in the tissue. Conversely, persistent loops are absent from trajectories that are not as spatiotemporally organized, while many non-persistent loops are observed, indicating a scattered point cloud devoid of any trajectory-like structure.

Finally, we compute Wasserstein distance to compare topological features across datasets (Fig. 1 K, step 4a). Intuitively, Wasserstein distance measures the cost of “transporting” the distribution of connected components and loops from one dataset to another. The distance is minimized when two datasets have identical *H0* and *H1* features, meaning that they have similar spatiotemporal signaling patterns. We provide a more detailed technical description of the Wasserstein metric in the Materials and methods section.

#### GSTH-based cell embeddings—Cell-specific heterogeneity in Ca^2+^ signaling (Fig. 1 K, bottom)

To understand differences in signaling between various cells, we compute scattering coefficients for each cell by aggregating over time in the scattering transform (Fig. 1 K, step 2b). We visualize the resulting GSTH cell embedding in 3D using PHATE, where each point in the PHATE plot corresponds to a single cell in the tissue (Fig. 1 K, step 3b). Two points are close together if the underlying Ca^2+^ signaling pattern for those cells is similar. For example, two cells spiking in phase will be close together compared with two cells spiking independently.

We quantify the difference in signaling activity between cell populations by measuring the proximity between the corresponding cells in the PHATE plot (Fig. 1 K, step 4b). Specifically, we estimate the relative likelihood of observing each cell type based on the sample density in the PHATE plot using MELD (Burkhardt et al., 2021). A relative likelihood close to 0.5 means that the cells are indistinguishable in terms of their signaling patterns, whereas values close to 0 or 1 mean that the cells have distinct signaling patterns.

### Ca^2+^ signaling is coordinated at long-range in the basal layer

**Figure S2.**
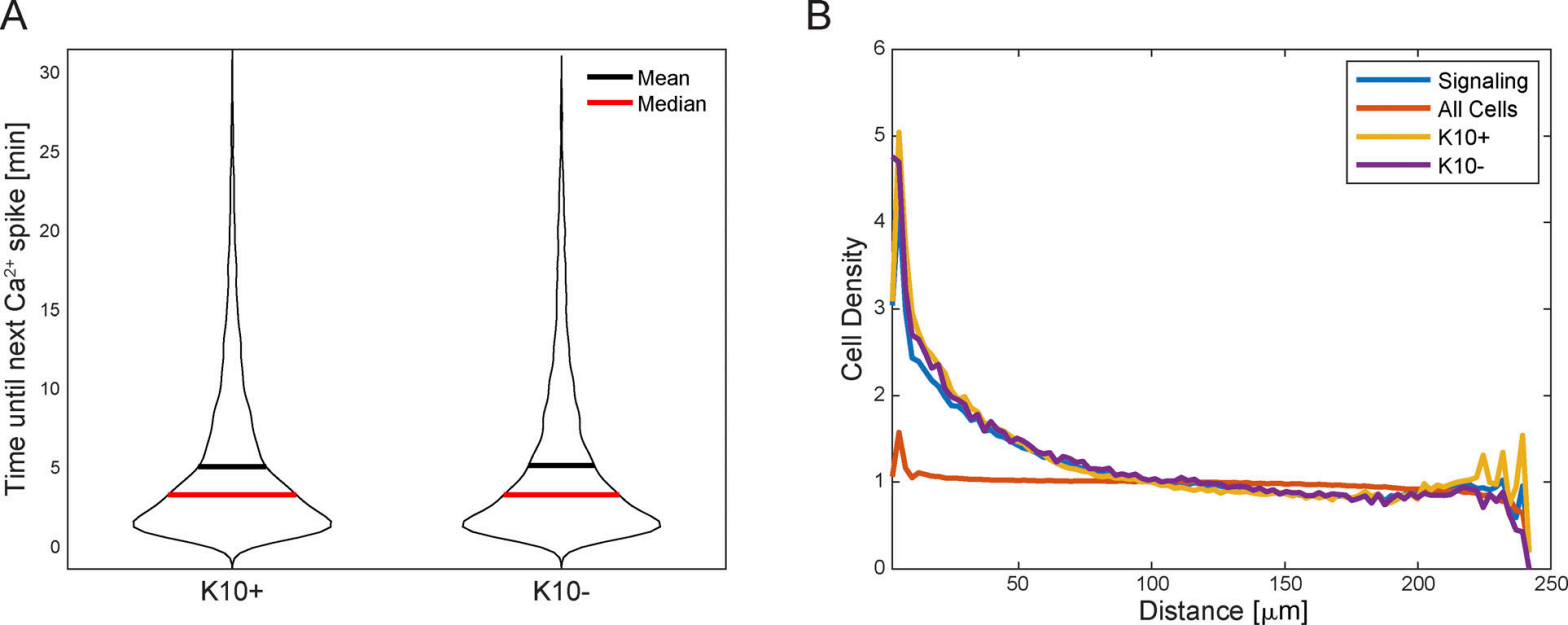
**K10+ and K10− populations do not show differences in some spatiotemporal signaling characteristics. (A)** Time interval between Ca^2+^ signal peaks for each cell (cells with one peak or less are ignored). ns = P = 0.9234, non-parametric Kruskal–Wallis test. **(B)** Average density of signaling cells around all cells (orange), signaling cells (blue), K10+ cells (yellow), or K10− cells (purple). The spatial distribution of all cells (signaling or not) is uniform. Signaling cells are clustered, but there is no significant difference between K10+ and K10− signaling cells. ns = P *=* 0.148, non-parametric Kruskal–Wallis test.

To further probe whether K10-reporter-positive (differentiating) cells displayed different spatiotemporal signaling characteristics than their K10-reporter-negative stem cell neighbors, we used our GSTH-based Cell Embeddings method (Fig. 1 K, bottom). With GSTH-based Cell Embeddings, each basal cell corresponds to a datapoint in the visualization, allowing us to directly compare the spatiotemporal characteristics of signaling across the thousands of cells within rather than between each recording. We applied this method to our K10-reporter datasets and colored each point in the PHATE plot based on each cell’s identity—either K10-reporter-positive or -negative (Fig. 1 L). We found that the K10-reporter-positive and -negative cell populations showed some overlap in their Ca^2+^ signaling patterns as indicated by MELD likelihood values close to 0.5, but that most points (cells) showed values close to 0 or 1 indicating distinct signaling dynamics in K10-reporter-positive compared to -negative cells (Fig. 1 M). The average time between Ca^2+^ spikes and the surrounding density of signaling cells were similar between the two populations (Fig. S2, A and B). Therefore, although we were unable to find clear differences in spatial or temporal signaling characteristics between K10-reporter-positive or -negative cells, our results comparing these populations with GSTH-based Cell Embeddings detected a complex spatiotemporal signaling pattern that is more common to K10-reporter-positive differentiation-committed progenitors than their K10-reporter-negative stem cell neighbors. Overall, our data demonstrate that both populations show similar Ca^2+^ signaling characteristics in many dimensions, and it is only once these cells leave the basal stem cell layer that they no longer display dynamic Ca^2+^ signaling.

**Figure S3.**
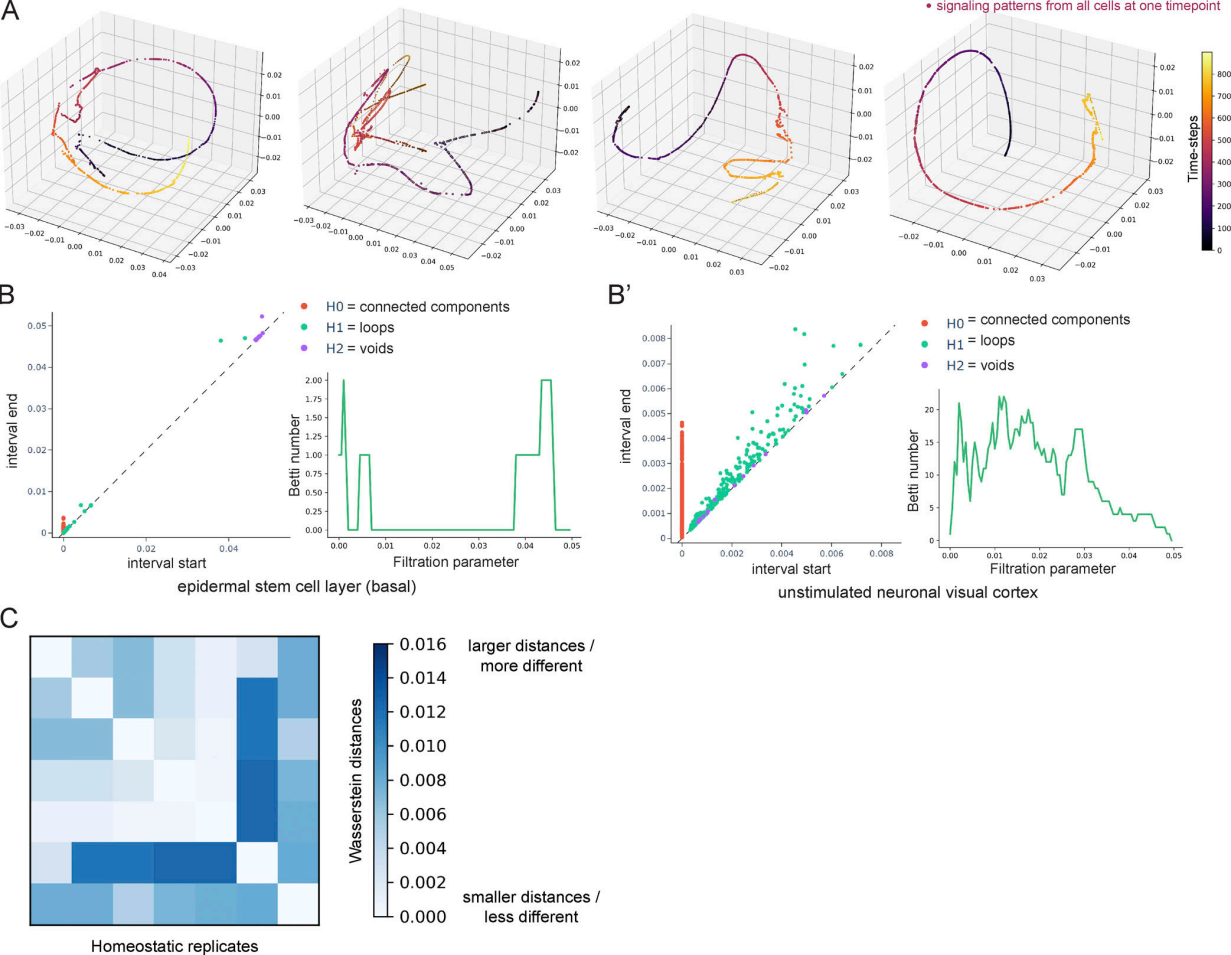
**Unsupervised modeling of Ca**^**2+**^ **signaling patterns reveals smooth, directed signaling in the homeostatic basal epidermis. (A)** PHATE visualizations of Ca^2+^ signaling time trajectories in the homeostatic basal epithelial layer from four 30-min timelapse movie replicates show smooth trajectories. **(B)** Persistence diagrams of Ca^2+^ signaling time trajectories in the quiescent neuronal visual cortex (right) versus the homeostatic epithelial stem cell layer (left). Each point corresponds to a topological feature in the trajectory, which appears at a certain interval start time and disappears at an interval end time, as data is gradually coarse-grained by merging nearby points across increasing levels of granularity. As an example, green points represent *H1* features that correspond to the formation of loops in the trajectory while purple dots represent *H2* features that correspond to the formation of voids. The further they are from the diagonals, the longer they exist, i.e., the larger their persistence. To the right, examples of corresponding Betti curves of *H1* loop features. The Betti numbers represent the number of loops found at increasing levels of granularity (as the filtration parameter increases). The epidermal stem cell layer shows loop features at large filtration values, whereas the dataset from the neuronal visual cortex shows more complexity and loops across all filtration parameters. **(C)** Heatmap of Wasserstein distances of *H0* features from seven timelapse movies (from at least three different mice) of the homeostatic epidermal stem cell layer, showing small differences in the signaling patterns across replicates.

Epithelial cells have been shown to display coordinated signaling across tissues in response to injury (Kumamoto et al., 2017; Isakson et al., 2001; Matsubayashi et al., 2004). Whether the local neighborhoods of Ca^2+^ signaling we observed here during homeostasis occur at random or are coordinated with one another is unclear. We applied GSTH (Fig. 1 K, top) to 30-min recordings (time-steps every 2 s) of homeostatic Ca^2+^ signaling. Each point in the PHATE time trajectory was color-coded based on time. Each point’s position in the 3D plot revealed the similarity or difference of the tissue’s Ca^2+^ signaling pattern from timepoint to timepoint. Our analyses of basal epidermal Ca^2+^ signaling recordings from many mice consistently produced smooth trajectories along timepoints, showing that Ca^2+^ transients steadily diffuse to neighboring cells in a directed and coordinated manner (Fig. 1 N and Fig. S3 A).

We next quantified the shape of the trajectories using the last step of GSTH, visualizing persistence diagrams and Betti curves of *H1* (loop) features (Fig. S3 B). Smooth trajectories create large-scale loops appearing later in the persistence diagram, whereas noisy trajectories with points deviating from the main trajectory will form persistence features appearing at earlier intervals. The epidermal layer persistence diagrams had a few noise features that quickly disappeared but mostly large-scale loops that appeared much later. We next quantified Wasserstein distances to compare topological features between all replicates from the epidermal stem cell layer. Small Wasserstein distances signify that two datasets have similar features and therefore similar spatiotemporal signaling patterns. The Wasserstein distances across all replicates were small (Fig. S3 C), signifying similar PHATE trajectories (hence similar signaling patterns). This analysis revealed an emergent property of this compartment that localized Ca^2+^ signals are coordinated and patterned in time and space across thousands of epithelial cells.

To determine how these consistently smooth PHATE trajectories from the regenerative epidermis compared with other tissues, we applied GSTH to a classic example of Ca^2+^ signaling in the nervous system using previously published recordings of Ca^2+^ signaling from 10,000 neurons of the primary visual cortex (Stringer et al., 2018, 2019). Spontaneous activity from the primary visual cortex has been shown not to be organized topographically during the resting state. Neurons are connected via long processes, so we used a correlation between neurons’ Ca^2+^ signals to create a cellular graph (instead of the nearest-neighbors graph built for epidermal cells). Our analysis with GSTH revealed markedly discontinuous, scattered time trajectories (Fig. 1 N’), indicating more abrupt changes in signaling patterns over time and less spatiotemporally coordinated signaling. To quantitatively compare the neuronal trajectory to the earlier epithelial stem cell layer datasets, we performed the last step of GSTH, visualizing persistence diagrams and Betti curves of *H1* (loop) features. In the neuronal trajectory, many features appeared and disappeared at all scales, revealing a complex data geometry (Fig. S3 B’), in contrast to the persistence diagram of the epidermal layer that had mostly large-scale loops at later intervals. The trajectories from the neuronal visual cortex were like the trajectories from our analysis of simulated data created using a uniform distribution, where we observed a very scattered time trajectory resembling a point cloud (Fig. 1 N”). Thus, our comparisons across different tissues and modalities highlight how the epidermal stem cell pool orchestrates tissue-wide coordinated Ca^2+^ signaling, demonstrating the spatial and temporal connectivity of information flow in the basal epithelium across multiple scales during homeostasis.

### G2 cells are essential for homeostatic Ca^2+^ signaling in the epidermal stem cell pool

**Figure 2.**
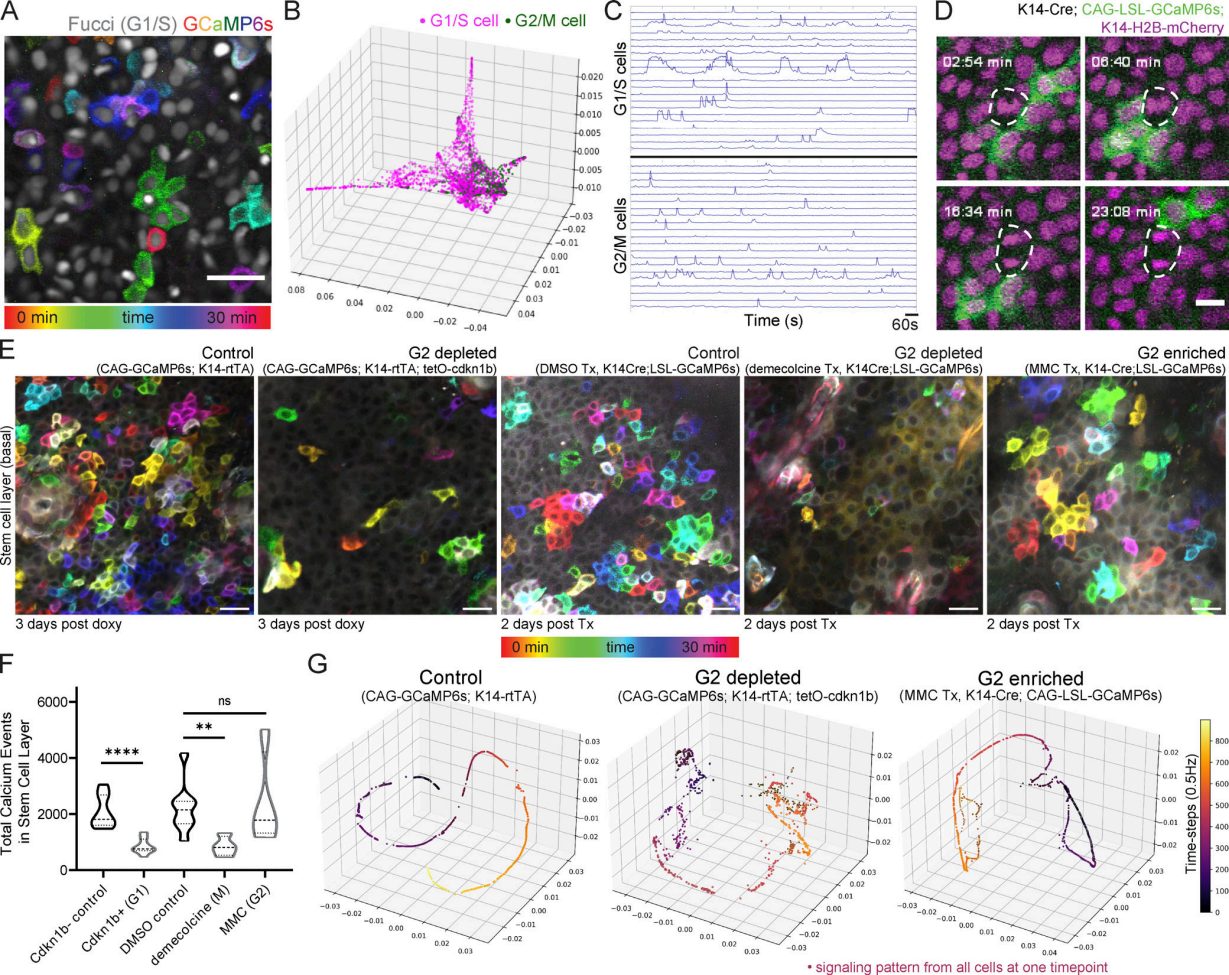
**G2 cells are necessary for Ca**^**2+**^ **signaling activity in the stem cell layer. (A)** Max intensity projection of 30-min recording of the epidermal basal layer of Ca^2+^-sensor mouse with a cell cycle reporter (*K14-Cre; CAG-LSL-GCaMP6s; R26p-Fucci2*). Only the mCherry-hCdt1 expression is visible marking G1 and S cells in grayscale. Color scale indicates GCaMP6s signal across time. Scale bar: 25 µm. **(B)** Representative PHATE plot of cell embeddings. Each dot represents a single cell; its position in space represents the similarity of its Ca^2+^ signaling pattern to other cells’ signaling; each cell/node is colored by its cell cycle state based on the thresholded Fucci signal. **(C)** Representative traces of Ca^2+^ activity (normalized GCaMP6s fluorescence intensity over time) in G1/S cells versus G2 cells. **(D)** Representative example of a mitotic cell with lack of Ca^2+^ signaling in live mice. Mitosing cells were identified via mitotic spindles via the nuclear H2B signal. Scale bar: 10 µm. **(E)** Representative max intensity projection of 30-min recording of the basal layer of control, G2-depleted, and G2-enriched Ca^2+^-sensor mice. Color scale indicates GCaMP6s signal across time. Cdkn1b mice imaged 3 d after induction. MMC- and demecolcine-treated mice imaged 2 d after treatment. Scale bar: 25 µm. **(F)** Total Ca^2+^ events identified in G2-depleted cdkn1b mice (stalled in G1), G2-enriched MMC-treated mice, and G2-depleted demecolcine-treated mice (stalled in mitosis) versus genetic or drug-treated controls. ****P *<* 0.0001, unpaired two-tailed Student’s *t* test, *n* = 8 control and 9 cdkn1b+ movies, **P *<* 0.01, one-way ANOVA, *n* = 8 DMSO control, 4 demecolcine, and 4 MMC movies. **(G)** PHATE time trajectory visualization of Ca^2+^ signaling in the cdkn1b+ G2-depleted basal layer and MMC-treated G2-enriched basal layer versus control basal layer. G2-depleted mice showed disruption of the smooth trajectories (representing loss of coordinated patterns of signaling), whereas the G2-enriched basal layer maintained homeostatic patterns.

**Figure S4.**
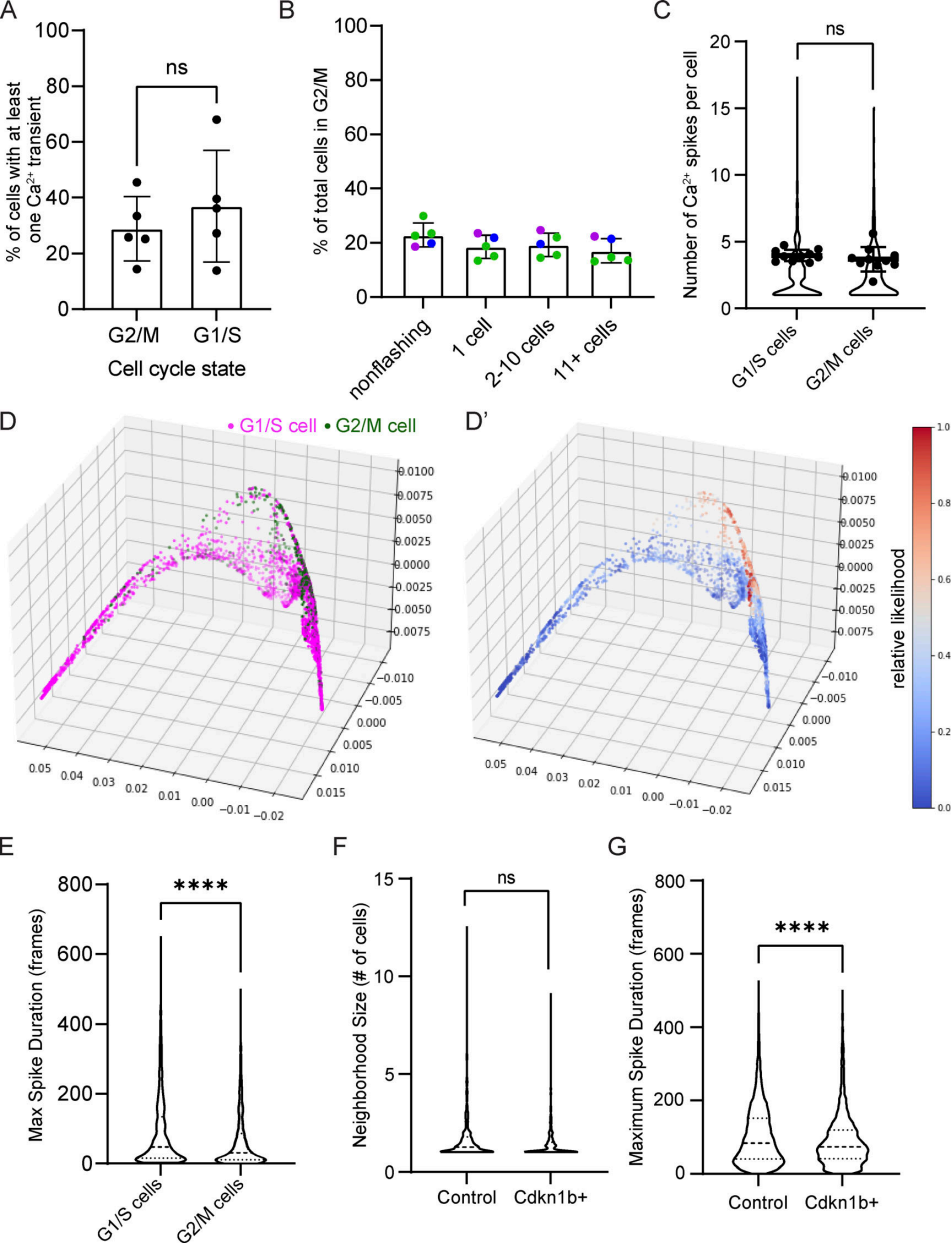
**Cell cycle–specific Ca**^**2+**^ **signaling. (A)** Percent of G1/S versus G2/M cells flashing over the course of 30 min. ns = P *>* 0.05, unpaired two-tailed Student’s *t* test, *n* = 5 30-min timelapse movies from three mice. **(B)** Percent of G2/M cells in groups of non-flashing, single flashing, small clusters, and large clusters of flashing cells based on mCherry-hCdt1 expression. ns = P *>* 0.05, one-way ANOVA with multiple comparisons, *n* = 5 30-min timelapse movies from three mice. **(C)** Number of Ca^2+^ spikes quantified per cell in G1/S cells versus G2/M cells. ns = P *>* 0.05, unpaired two-tailed Student’s *t* test, *n* = 10 timelapse movies from four individual mice. **(D)** PHATE visualization of cell embeddings of Ca^2+^ signaling patterns, where each dot represents a single cell; its position in space represents how similar its Ca^2+^ signaling is to other cells in space; each cell or node is colored by its cell cycle state based on nuclear Fucci2 signal. **(D’)** MELD comparison of G1/S versus G2/M signaling patterns. Values close to 0.5 represent the most overlap between the two populations. **(E)** Maximal spike duration (maximum number of frames between the start and end of individual Ca^2+^ events) in G1/S versus G2/M cells. P *<* 0.0001, Mann–Whitney test, *n* = 5,218 G1/S cells and 1,040 G2/M cells from 10 timelapse movies. **(F)** Frequency of different neighborhood sizes of spatiotemporally connected Ca^2+^ signaling from 30-min timelapses of cdkn1b+ G1-stalled basal layers versus control in Ca^2+^ sensor mice. ns = P *>* 0.05, unpaired two-tailed Student’s *t* test, *n* = 9 control and 8 Cdkn1b+ timelapse recordings. **(G)** Maximal spike duration in control versus G1-stalled cdkn1b+ mice. P *<* 0.0001, Mann–Whitney test, *n* = 7,184 cells from 9 control timelapse recordings and 2,470 cells from 8 cdkn1b+ timelapse recordings.

We show that Ca^2+^ signaling events are coordinated across large regions of the basal stem cell compartment. The basal stem cell layer of the epidermis is a dynamic environment characterized by a heterogeneous distribution of cells in various phases of the cell cycle (Hiratsuka et al., 2015). We therefore wondered whether cells in different cell cycle stages might show distinct spatiotemporal patterns, like K10-reporter-positive versus -negative populations (only about 10–15% of cycling S/G2/M cells will have K10 protein; Cockburn et al., 2022). To address this, we used the Ca^2+^ sensor combined with the Fucci cell cycle reporter that fluorescently labels G1 and S cells in red (Abe et al., 2013). In our system, Fucci negative cells represent G2 or mitotic cells, allowing us to compare the two halves of the cell cycle. We observed clusters of Ca^2+^ transients propagating across cells of G1/S and G2/M cell cycle stages (Fig. 2 A) with similar overall Ca^2+^ signaling activity between cell cycle states (Fig. S4, A–C).

To investigate the spatiotemporal characteristics of Ca^2+^ signaling across cells of different cell cycle stages, we again employed the GSTH-based Cell Embeddings method described above. Here, we colored each point in the PHATE plot based on its cell cycle stage to compare G1/S versus G2/M cells in terms of their Ca^2+^ signaling patterns in 3D space (Fig. 2 B and Fig. S4 D). We did not see a high degree of overlap between the two populations, as indicated by MELD likelihood values far from 0.5 (Fig. S4 D’). We found that G2/M cells clustered together, showing related Ca^2+^ signaling patterns, whereas G1/S cells were highly dispersed across the PHATE plots, indicating heterogeneous signaling patterns. Noticeably, G2/M cells displayed more homogeneous Ca^2+^ signaling patterns, exhibiting many short Ca^2+^ transients (Fig. 2 C and Fig. S4 E). These results demonstrate that G2/M cells display Ca^2+^ signaling patterns that are more similar to each other in spatial and temporal dimensions than G1 or S cells.

To better resolve the Ca^2+^ signaling dynamics of G2 versus M phase cells, we labeled all nuclei (*K14-Cre; R26-CAG-LSL-GCaMP6s; K14-H2B-mCherry*) and tracked mitotic events as they occurred, while also recording Ca^2+^ signaling. Surprisingly, while extensive literature links transient elevations in Ca^2+^ with important steps of mitosis (Ratan et al., 1988; Kao et al., 1990; Poenie et al., 1985), we found that cells undergoing mitosis do not display intracellular Ca^2+^ transients during homeostasis (Fig. 2 D and Video 2). Together, these data illustrate that G2 cells display spatiotemporally similar patterns of short Ca^2+^ spikes, distinct from the very heterogeneous signaling patterns of G1 and S cells and the non-signaling M cells.

**Video 2.** **Mitotic cells do not participate in homeostatic Ca**^**2+**^ **signaling.** Intravital imaging of Ca^2+^ sensor mice (*K14-Cre; R26-CAG-LSL-GCaMP6s; K14-H2B-mCherry*) with eight examples of basal epithelial cells undergoing mitosis showing a lack of Ca^2+^ signaling. Magenta marks the nuclei of each cell and green fluorescence intensity represents relative cytosolic Ca^2+^ levels. Frames were taken every 2 s for a total duration of 30 min. Movie shows dynamics at 60 frames per second or 120× the actual timescale. Scale bar: 10 μm.

**Figure S5.**
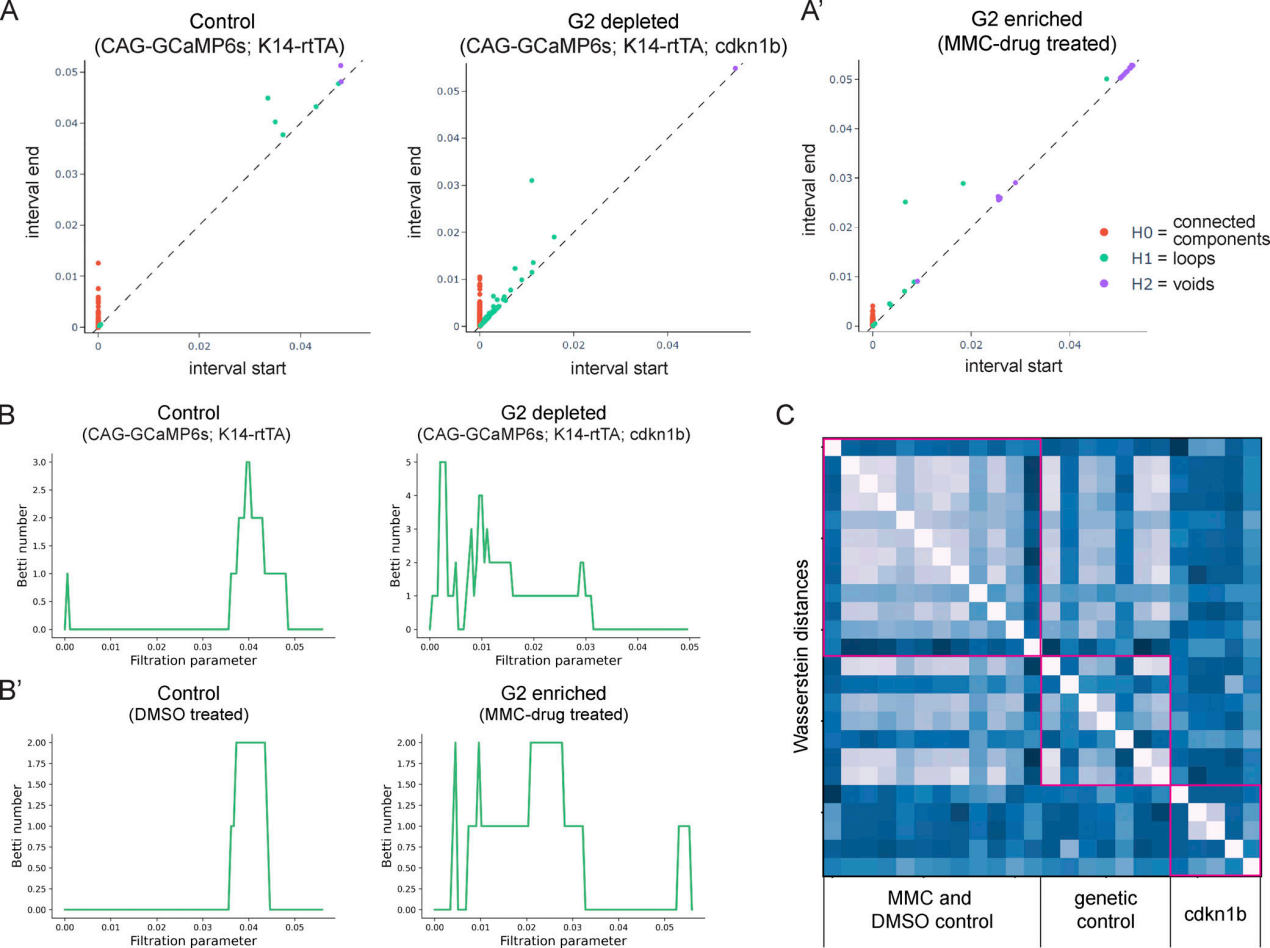
**Enrichment of G2 cells uniquely does not disrupt coordinated Ca**^**2+**^ **signaling. (A and A’)** Representative persistence diagrams (*H0*: connected components, *H1*: loops, *H2*: voids) for G2-depleted (A) and G2-enriched (A’) conditions (*CAG-GCaMP6s; K14-rtTA; cdkn1b* 3 d after induction and *K14-Cre; CAG-LSL-GCaMP6s* 2 d after MMC drug treatment, respectively). The persistence diagram from the G1-stalled group has earlier *H1* (loop) features, indicating scattered trajectories and less coordinated signaling. **(B)** Representative Betti curves of *H1* features (loops) for G1 and G2 enriched conditions (*CAG-GCaMP6s; K14-rtTA; cdkn1b* 3 d after doxycycline treatment and *K14-Cre; CAG-LSL-GCaMP6s* 2 d after MMC drug treatment, respectively). **(C)** Heatmap of the Wasserstein distances from the persistence diagrams of G2-enriched MMC, DMSO control, G1-stalled Cdkn1b, and genetic control mice. Distances are small and similar across G2-enriched and all control mice and larger and different when compared to G1-stalled mice.

We next questioned whether G2 cells are functionally important in the regulation of Ca^2+^ signaling activity by both depleting and enriching for G2 cells within the basal stem cell layer. First, we depleted G2 phases by stalling cells in the G1 state, using the keratin 14 promoter to induce the cell cycle inhibitor Cdkn1b (p27) in epidermal basal cells, and combined this with a constitutive Ca^2+^-sensor (*K14-rtTA*; *tetO-Cdkn1b; Rosa26-CAG-GCaMP6s*). Through timelapse recordings, we observed a marked decrease in the total amount of Ca^2+^ signaling in G2-depleted Cdkn1b mice compared with littermate controls (Fig. 2, E and F; and Video 3). We saw no change in the neighborhood size of clustered Ca^2+^ signaling upon cell cycle inhibition (Fig. S4 G); however, our analyses revealed a temporal disruption, such that cells exhibited shorter Ca^2+^ spikes (Fig. S4 H). Comparison between G2-depleted and control cells at a population level via GSTH analysis also revealed different patterns between the two groups (Fig. 2 G). The PHATE trajectories for the control group were smooth, consistent with coordinated signaling in wildtype tissue; however, trajectories for the G2-depleted Cdkn1b mice showed scattered patterns. These characteristics were also quantified in the persistence diagrams and the corresponding Betti curves of *H1* features (Fig. S5, A and B). Betti curves for the G2-depleted group showed that loops were formed and closed at earlier thresholds, reflecting scattered time trajectories and disrupted signaling coordination. We also depleted G2 cells by treating mice with the drug demecolcine to stall basal cells in mitosis. Similar to the depletion of G2 cells in the Cdkn1b condition, we found that Ca^2+^ signals were abrogated with demecolcine treatment (Fig. 2, E and F; and Video 4). These data demonstrate that a homogeneous layer of G2-depleted cells is not able to initiate coordinated Ca^2+^ signaling.

**Video 3.** **Basal cells stalled in G1 of their cell cycle show low levels of Ca**^**2+**^ **signaling.** Disrupted Ca^2+^ dynamics in G2-depleted Cdkn1b mice (K14-rtTA; tetO-Cdkn1b; R26-GCaMP6s) on the right compared to littermate controls (K14-rtTA; R26-GCaMP6s) on the left, 3 d after start of doxycycline administration. Green fluorescence intensity represents relative cytosolic Ca^2+^ levels. Frames were taken every 2 s for a total duration of 30 min. Movie shows dynamics at 40 frames per second or 80× the actual timescale. Scale bar: 25 μm.

**Video 4.** **Basal cells stalled in mitosis show low levels of Ca**^**2+**^ **signaling.** Disrupted Ca^2+^ dynamics in G2-depleted demecolcine-treated mice on the right compared to DMSO vehicle-treated littermate controls on the left, 2 d after drug treatment. Magenta marks the nuclei of each cell and green fluorescence intensity represents relative cytosolic Ca^2+^ levels. Frames were taken every 2 s for a total duration of 23 min. Movie shows dynamics at 40 frames per second or 80× the actual timescale. Scale bar: 25 μm.

We next tested the opposite scenario and enriched for G2 cells by treating mice with the drug Mitomycin C (MMC). Unlike mice enriched for G1 or mitotic cells, we observed normal spatiotemporal patterns of Ca^2+^ signaling in G2-enriched mice, similar to DMSO vehicle-treated controls (Fig. 2, E and F; and Video 5). GSTH also revealed smooth PHATE trajectories (Fig. 2 G). To quantitatively compare the topology of the PHATE plots of G2-enriched signaling, we plotted persistence diagrams for each PHATE time trajectory (Fig. S5, A’ and B’). The G2-enriched datasets showed *H1* features (loops) were formed and closed at later thresholds, reflecting the smoothness of the time trajectory and spatiotemporal coordination of the signaling, like the controls. We next quantified Wasserstein distances (a way to measure differences in topology between trajectories) between the MMC, Cdkn1b, and control trajectories (Fig. S5 C). The Wasserstein distances within the MMC G2-enriched and DMSO control groups were small, signifying similar PHATE trajectories (hence similar signaling patterns). However, the Wassertein distances between the Cdkn1b G2-depleted and genetic control groups were large, indicating different signaling patterns. These data demonstrate that the epidermal stem cell pool requires G2 cells to generate normal levels of coordinated Ca^2+^ signaling across the epidermal stem cell layer.

**Video 5.** **Basal cells stalled in G2 of their cell cycle show normal patterns of Ca**^**2+**^ **signaling.** G2-enriched MMC-treated mice on the right compared to DMSO vehicle-treated littermate controls on the left, 2 d after drug treatment. Magenta marks the nuclei of each basal cell and green fluorescence intensity represents relative cytosolic Ca^2+^ levels. Frames were taken every 2 s for a total duration of 30 min. Movie shows dynamics at 40 frames per second or 80× the actual timescale. Scale bar: 25 μm.

**Figure S6.**
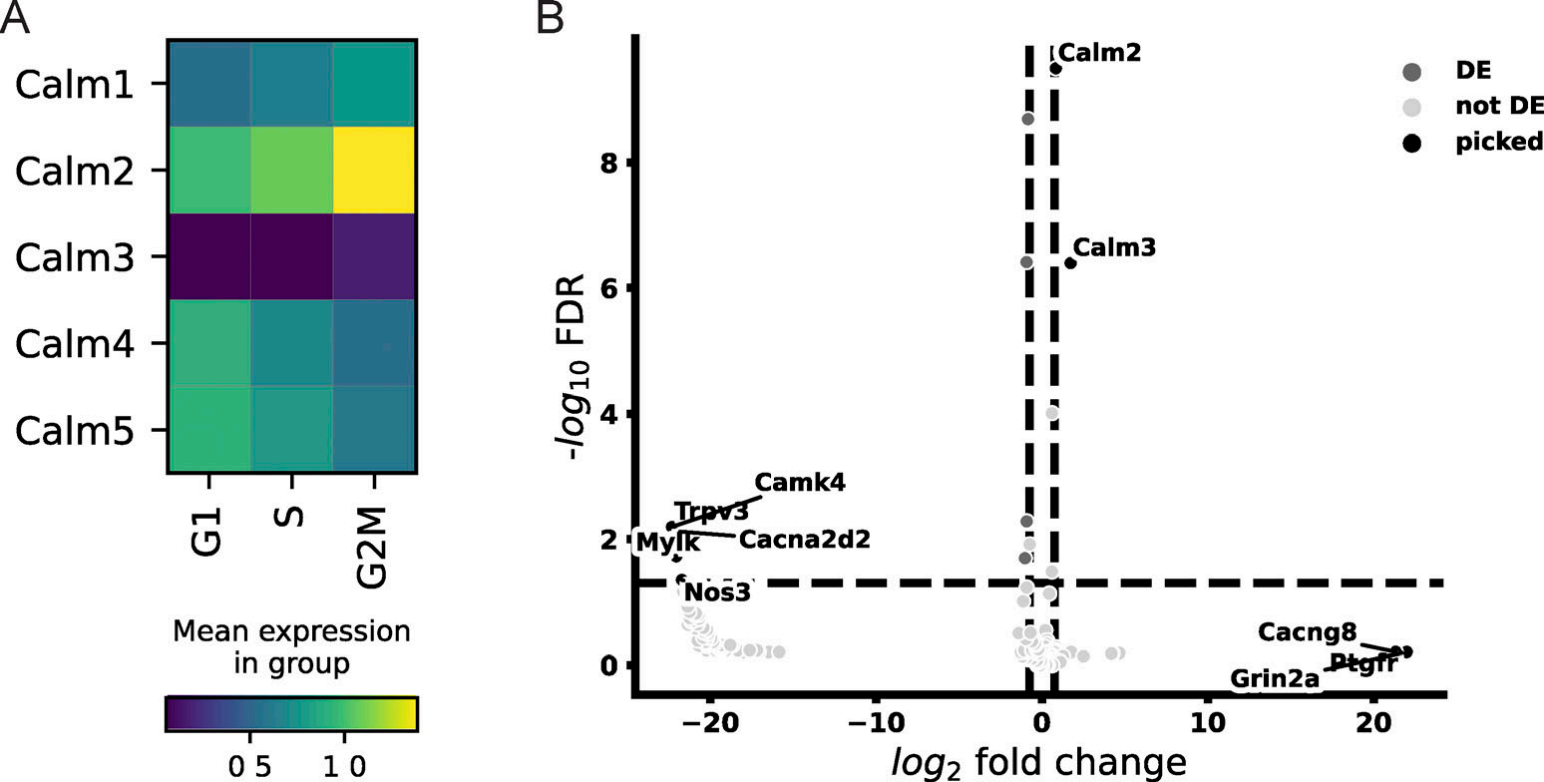
**G2 cells show differential transcription of key Ca**^2+^ **signaling genes. (A)** Heatmap of gene expression patterns of Calmodulin genes showing changes of epidermal basal cells during cell cycle progression. Expression levels are shown as average of log-normalized expression. **(B)** Volcano plot showing differentially expressed Ca^2+^ signaling genes in G1/S versus G2/M cells. DE, differentially expressed; picked, top 10 genes.

We wondered what molecular differences could characterize G2 cells and allow them to regulate Ca^2+^ signaling in this way. As a starting point, we analyzed single-cell RNA sequencing data (Cockburn et al., 2022) to compare gene expression of Ca^2+^ signaling pathway genes between different cell cycle stages in epidermal basal cells. We found that, of 232 Ca^2+^ signaling pathway genes, *Calm2* was the most prominently upregulated Ca^2+^ signaling pathway gene in G2/M cells, which could be an interesting target for further investigation (Fig. S6, A and B; and Table S1). Together, these results reveal that G2 cells carry out Ca^2+^ dynamics with more uniform spatiotemporal characteristics and that G2 cells are essential for coordinated Ca^2+^ signaling activity in the stem cell compartment.

### Cx43 orchestrates Ca^2+^ signaling at large scales, but not within local neighborhoods

**Figure 3.**
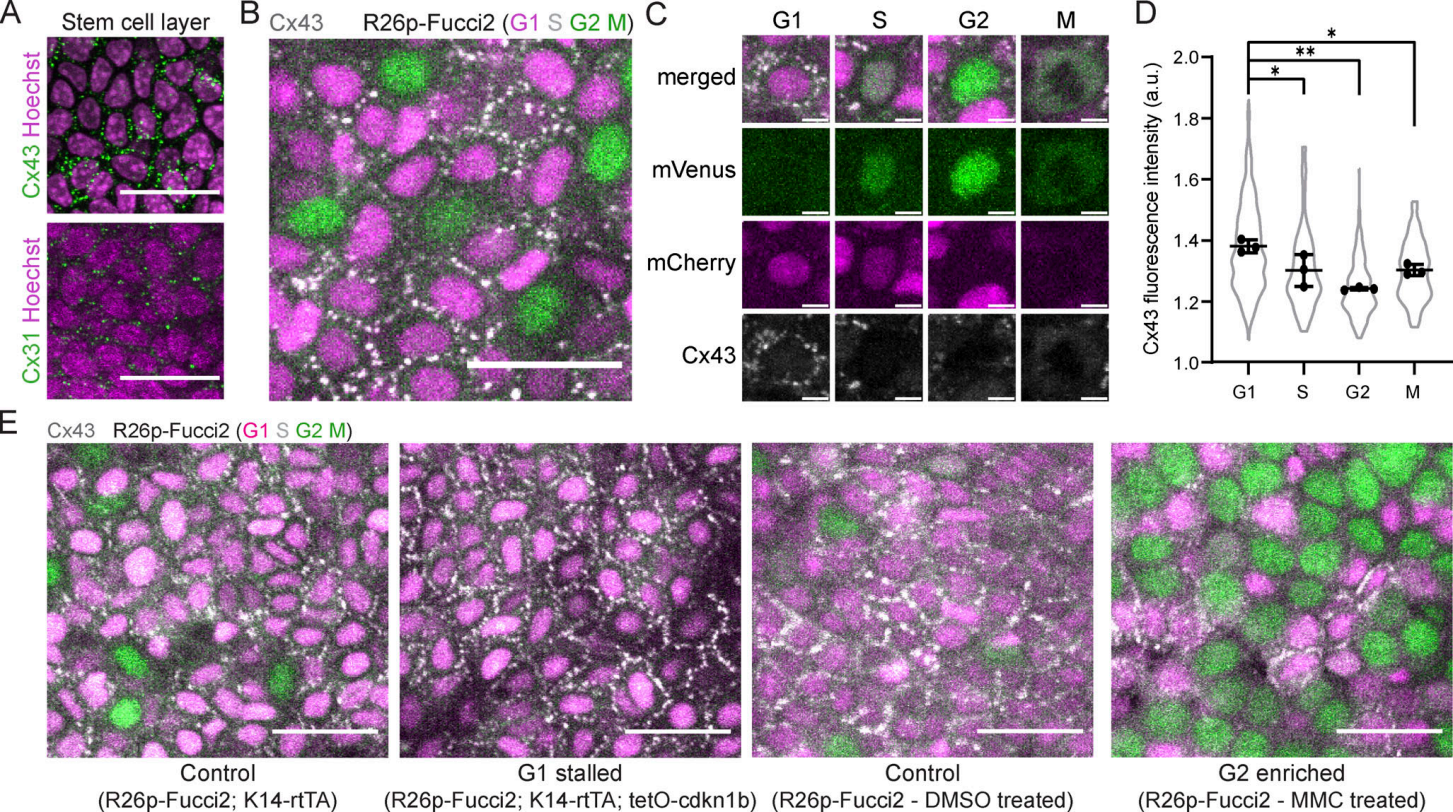
**Cx43 distribution at junctions is enriched in G1 basal stem cells. (A)** Maximum intensity projection of whole-mount immunofluorescence staining for Cx43 (top in green) and Cx31 (bottom in green) in the basal stem cell layer (Hoechst marking nuclei in magenta). Scale bar: 25 µm. **(B)** Maximum intensity projection of whole-mount immunofluorescence staining for Cx43, shown in white, in *R26p-Fucci2* mice, where G1 and S cells are mCherry^+^ (magenta) and S, G2, and M cells are mVenus^+^ (green). Scale bar: 25 µm. **(C)** Insets of G1, S, G2, and M cells in Rosa26p-Fucci2 mice with Cx43 immunofluorescence staining shown in white. Scale bar: 5 µm. **(D)** Quantification of Cx43 mean fluorescence intensity at the borders of G1, S, G2, and M cells in Rosa26p-Fucci2 mice. *P *<* 0.05, **P *<* 0.01, one-way ANOVA, *n* = 3 control and 3 Cx43 cKO mice. **(E)** Cx43 whole-mount immunofluorescence staining (white) in G1- and G2-enriched Rosa26p-Fucci2 mice, where G1 and S cells are mCherry^+^ (magenta) and S, G2, and M cells are mVenus^+^ (green). G2 depleted and control (*K14-rtTA; tetO-cdkn1b; R26p-Fucci2* and *K14-rtTA; R26p-Fucci2*) tissue was collected 3 d after induction. G2-enriched MMC-treated and DMSO-treated control *R26p-Fucci2* tissue was collected 2 d after treatment. Scale bar: 25 µm.

Our findings have established a role for the cell cycle phase in the regulation of Ca^2+^ signaling, yet we lack an understanding of the molecular mediators for long-range coordination of Ca^2+^ dynamics during epidermal regeneration. Gap junctions are known mediators of Ca^2+^ signaling in epithelial tissues, directly linking the cytoplasm of neighboring cells (Laird, 2010; Churko and Laird, 2011). To determine how cells within the basal stem cell layer are connected to their neighbors via gap junctions, we stained for connexin 31 (Cx31) and Cx43, the two most highly expressed connexins in this layer (Kretz et al., 2003; Martin and van Steensel, 2015). We observed high levels of Cx43 gap junctions compared with Cx31 (Fig. 3 A). Interestingly, we found that the localization of Cx43 gap junctions to cellular junctions was highly heterogeneous from cell to cell. We examined Cx43 distribution via immunofluorescence at different stages of the cell cycle in the homeostatic epidermis of cell cycle reporter mice (*R26p-Fucci2*) and found that stem cells display a gradient of junctional Cx43 expression as they pass through different cell cycle phases, with maximal junctional localization in the G1 stage (Fig. 3, B–D). These data show that Cx43 is dynamically regulated throughout the cell cycle and prompted us to interrogate Cx43 localization in the genetic models and drug treatments from our earlier experiments. Enrichment or depletion of G1 cells confirmed that Cx43 is most highly localized to cell–cell junctions in the G1 population (Fig. 3 E) and that Cx43^low^ G2 cells can carry out homeostatic Ca^2+^ signaling patterns whether or not Cx43^high^ G1 cells were present, suggesting that G2-specific Ca^2+^ signaling proteins or other connexins could play a role in coordinating Ca^2+^ signaling in G2 cells.

**Figure S7.**
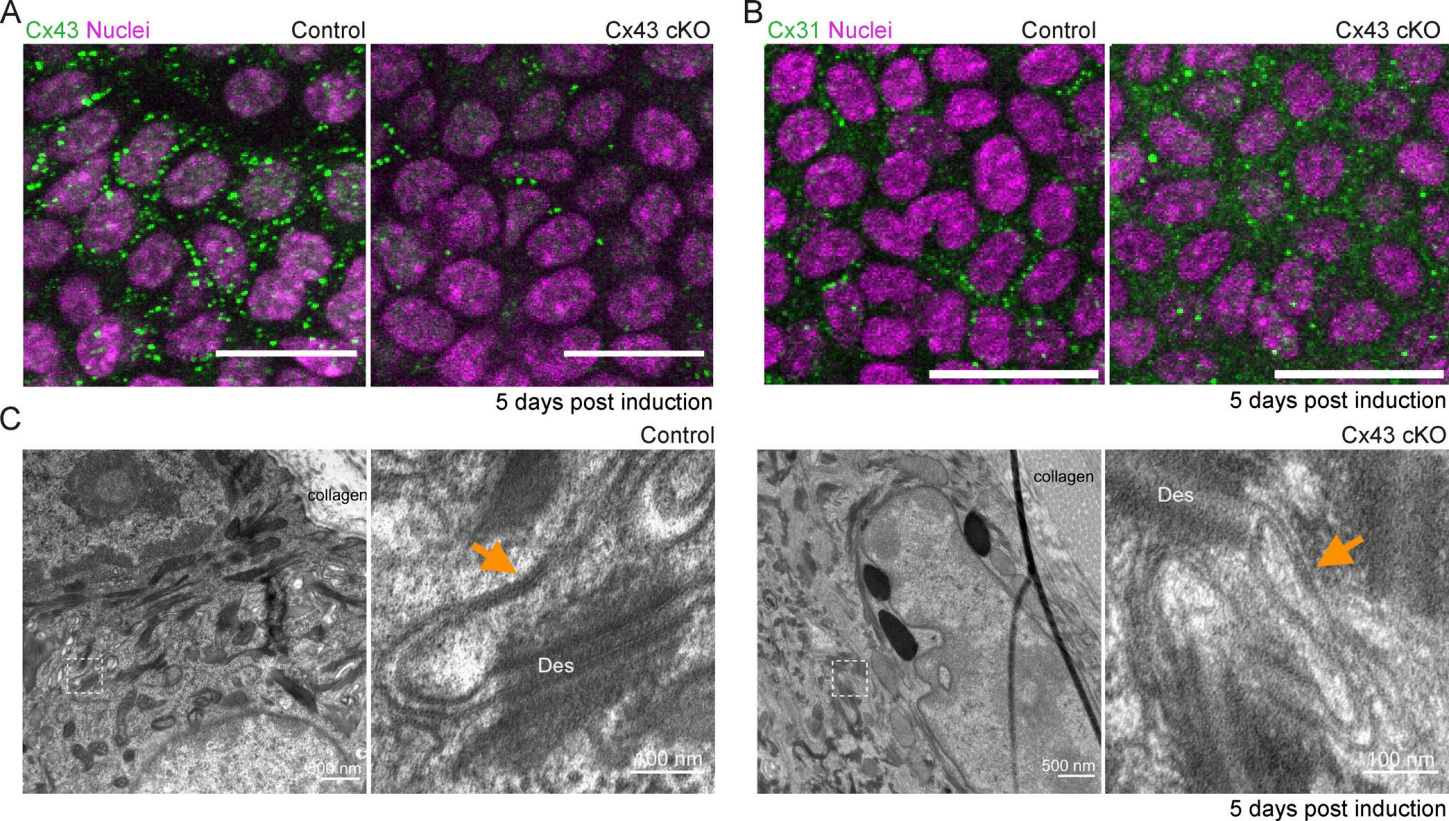
**Loss of Cx43 does not fully disrupt all gap junctions or the integrity of the tissue. (A)** Immunofluorescence staining of basal layer from *K14-CreER; Cx43*^*+/+*^ and *K14-CreER; Cx43*^*fl/fl*^ mice 5 d after tamoxifen induction. Cx43 in green and DAPI (nuclei) in magenta. Scale bar: 25 µm. **(B)** Immunofluorescence staining of epidermal basal stem cell layer from *K14-CreER; Cx43*^*+/+*^ and *K14-CreER; Cx43*^*fl/fl*^ mice 5 d after tamoxifen induction, with staining for Cx31 in green and Hoechst marking nuclei in magenta. Scale bar: 25 µm. **(C)** Gap junctions within the epidermal basal layer of *K14-CreER; Cx43*^*+/+*^ and *K14-CreER; Cx43*^*fl/fl*^ mice 5 d after tamoxifen induction imaged by transmission electron microscopy (TEM). Inset shows a higher magnification view of gap junctions (orange arrowheads).

Given that Cx43 is highly expressed in most cells in the basal epidermal layer, we next asked whether Cx43 is essential for homeostatic patterns of Ca^2+^ signaling. To address this, we crossed Cx43 conditional knockout mice (cKO) with a germline recombined Ca^2+^-sensor line (*K14-CreER; Cx43^fl/fl^; Rosa26-CAG-GCaMP6s* and *K14-CreER; Cx43*^*+/+*^; *Rosa26-CAG-GCaMP6s* littermate controls) and performed live timelapse imaging at 1, 5, and 7 d after induction. We first confirmed loss of Cx43 protein expression within 5 d of recombination (Fig. S7 A). While loss of Cx43 abolished Cx43 gap junctions, it did not abolish all gap junctions, as detected by immunofluorescence whole-mount staining for Cx31 and electron microscopy (Fig. S7, B and C).

**Figure 4.**
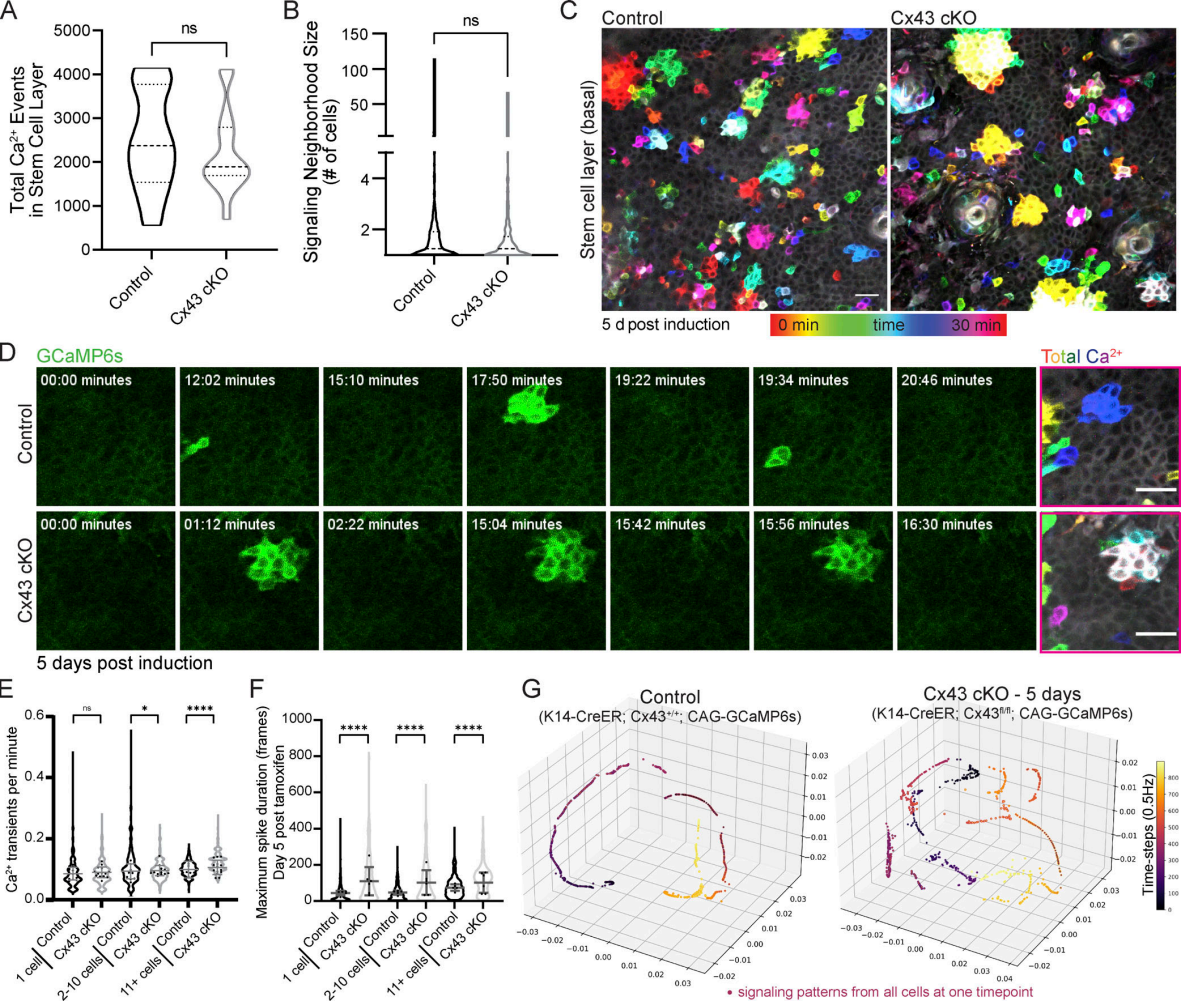
**Cx43 is necessary for long-range Ca**^**2+**^ **signaling coordination but not local signaling activity. (A)** Total number of Ca^2+^ signaling events in control versus Cx43 cKO mice. ns = P *>* 0.05, unpaired two-tailed Student’s *t* test, *n* = 11 (control) and 14 (Cx43 cKO) recordings from at least three mice per condition. **(B)** Average neighborhood size of signaling in control versus Cx43 cKO mice. ns = P *>* 0.05, unpaired two-tailed Student’s *t* test, *n* = 11 (control) and 14 (Cx43 cKO) recordings from at least three mice per condition. **(C)** Max intensity projection of 30-min recordings of the stem cell pool of control and Cx43 cKO Ca^2+^-sensor mice 5 d after induction (*Rosa26-CAG-GCaMP6s; K14-CreER; Cx43*^*+/+*^ and *Rosa26-CAG-GCaMP6s; K14-CreER; Cx43*^*fl/fl*^). Color scale represents time. Repeated signaling manifests as a white signal (the sum of colors). Scale bar: 25 µm. **(D)** Time-course of clustered signaling from 30-min movies of the stem cell pool of control and Cx43 cKO Ca^2+^-sensor mice 5 d after induction. Last image on right is max intensity projection with time represented by a color scale. Scale bar: 25 µm. **(E)** Ca^2+^ transients per minute per cell for three patterns of Ca^2+^ signaling (1 cell, 2–10 cells, or 11+ cells) in control versus Cx43 cKO Ca^2+^-sensor mice. ns = P *>* 0.05, *P = 0.0139, ****P *<* 0.0001, Mann–Whitney test. **(F)** Maximal spike duration of Ca^2+^ transients per cell for three patterns of Ca^2+^ signaling (1 cell, 2–10 cells, or 11+ cells) in control versus Cx43 cKO mice 5 d after tamoxifen induction. ****P *<* 0.0001, Mann–Whitney test. **(G)** Representative PHATE visualization of Ca^2+^ signaling in the Cx43 cKO versus control stem cell pool shows disruption of smooth, directed, and coordinated patterns of signaling in mice 5 d after loss of Cx43.

Surprisingly, we observed no change in the average number of Ca^2+^ events or the distribution of signaling neighborhood sizes upon loss of Cx43 (Fig. 4, A and B). Temporal color coding frames of the recordings from the Cx43 cKO mice revealed that Ca^2+^ signaling neighborhoods were more spatially restricted and signaled more repeatedly in contrast to more dispersed and heterogeneous spatial patterns of Ca^2+^ signaling in the littermate controls (Fig. 4, C and D; and Video 6). Reflecting this spatial restriction, we observed an increased frequency of clustered Ca^2+^ signaling in Cx43 cKO mice (Fig. 4 E), and loss of Cx43 resulted in a longer maximal spike duration for transients of Ca^2+^ signaling across all neighborhood sizes, most dramatically in single cells and small neighborhoods of 2–10 cells (Fig. 4 F). While local spread of Ca^2+^ signals was not abolished, disruption in the spatiotemporal characteristics of local neighborhoods of Ca^2+^ signaling demonstrated a role for Cx43 in regulating spatiotemporal Ca^2+^ signaling dynamics and prompted us to question whether loss of Cx43 affects signaling coordination at a tissue-wide level.

**Video 6.** **Cx43 orchestrates Ca**^**2+**^ **signaling at large scales, but not across local neighborhoods, in the stem cell pool.** Disrupted Ca^2+^ dynamics in Cx43 cKO mice (*K14CreER; Cx43*^*fl/fl*^*; R26-GCaMP6s*) on the right compared to littermate controls (*K14CreER; Cx43*^*+/+*^*; R26-GCaMP6s*) on the left, 5 d after tamoxifen induction. Green fluorescence intensity represents relative cytosolic Ca^2+^ levels. Frames were taken every 2 s for a total duration of 30 min. Movie shows dynamics at 40 frames per second or 80× the actual timescale. Scale bar: 25 μm.

**Figure S8.**
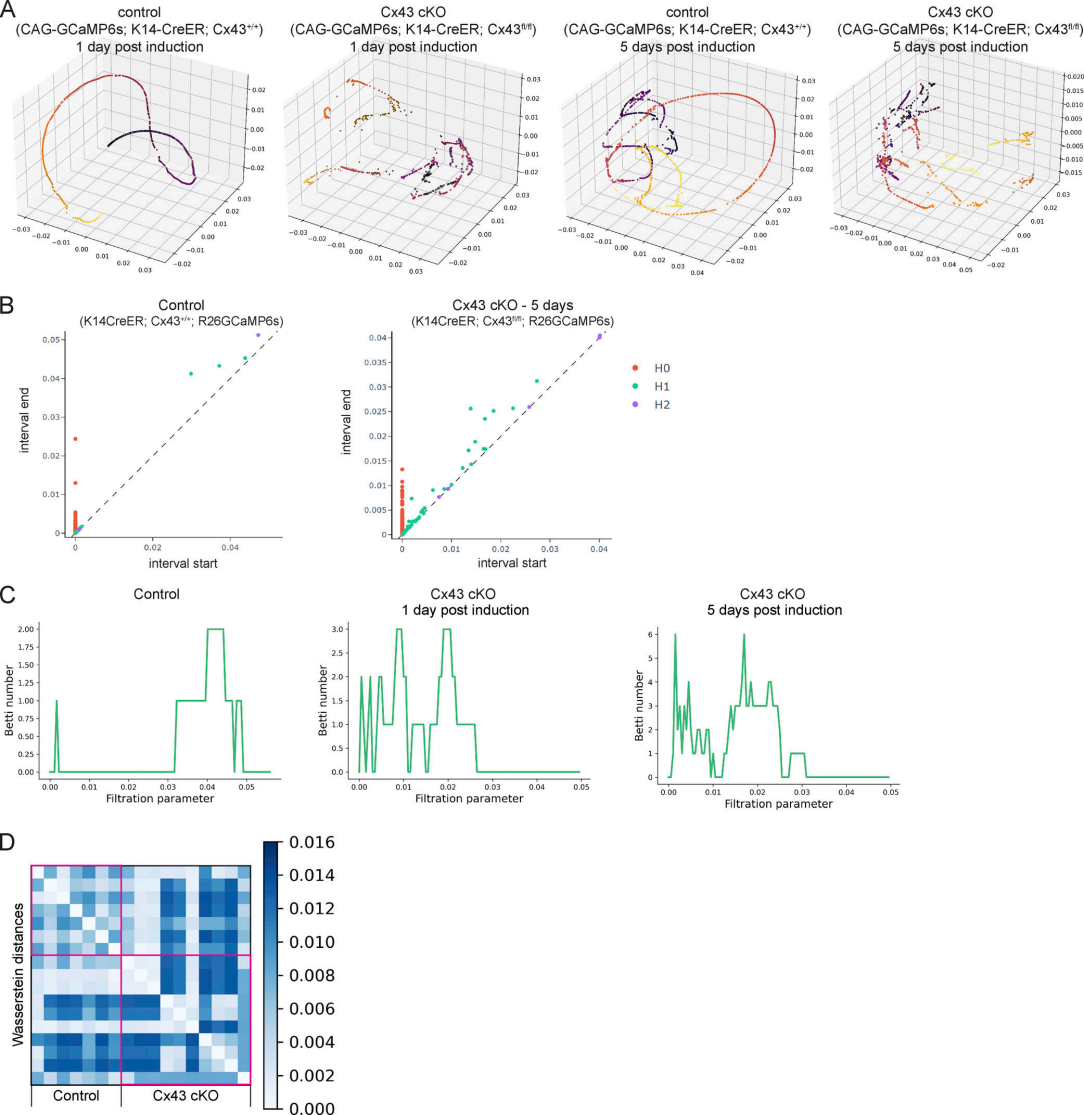
**Loss of Cx43 disrupts coordinated Ca**^**2+**^ **signaling patterns. (A)** Further PHATE visualizations of Ca^2+^ signaling time trajectories in the Cx43 conditional knockout versus control basal layer show disruption of smooth, directed, and coordinated patterns of signaling in mice 1 and 5 d after loss of Cx43. **(B)** Representative persistence diagrams (*H0*: connected components, *H1*: loops, *H2*: voids) for control and Cx43 cKO mice (*Rosa26-CAG-GCaMP6s; K14-CreER; Cx43*^*+/+*^ and *Rosa26-CAG-GCaMP6s; K14-CreER; Cx43*^*fl/fl*^) 5 d after induction. *H1* features from Cx43 cKO mice appear later in time and have a longer persistence. **(C)** Representative Betti curves of *H1* features (loops) for control and Cx43 cKO mice (*Rosa26-CAG-GCaMP6s; K14-CreER; Cx43*^*+/+*^ and *Rosa26-CAG-GCaMP6s; K14-CreER; Cx43*^*fl/fl*^) 1 and 5 d after tamoxifen induction. **(D)** Heatmap of Wasserstein distances of *H0* features from timelapse movies of the epidermal stem cell layer in Cx43 cKO versus control mice, showing larger distances when comparing cKO with wildtype controls.

To address this, we applied GSTH to 30-min recordings from Cx43 cKO and control mice. We observed a striking loss of smooth, coordinated Ca^2+^ signaling time trajectories in the epidermal stem cell pool upon loss of Cx43 compared with littermate control mice (Fig. 4 G and Fig. S8 A). Instead, Ca^2+^ signaling trajectories in the Cx43 mutant mice appeared more scattered and rougher, showing more rapid changes of signals over the graph and less connected neighborhoods of intercellular signaling. This was also quantified and reflected by the persistence diagrams of each trajectory (Fig. S8 B). As a reminder, in persistence diagrams, *H0* features represent connected components and *H1* features represent loops. Most loop *H1* features in the Cx43 cKO group appeared and disappeared at earlier stages than in the controls, reflecting rough trajectories (Fig. S8 C). Finally, we compared overall Ca^2+^ signaling patterns by quantifying Wasserstein distances between the persistence homology plots of Cx43 cKO and control populations and found that the distances were large, indicating different patterns (Fig. S8 D), further demonstrating that loss of Cx43 disrupts long-range signaling coordination observed at homeostasis. Perturbed trajectories across the stem cell pool were evident as early as 1 d after the loss of Cx43, suggesting a direct role for Cx43 in Ca^2+^ signaling regulation. The basal layer is a mix of cells in different cell cycle stages, and G1 cells make up the majority of the stem cell layer. We showed earlier that G2 cells are necessary for proper levels of Ca^2+^ signaling activity and that G2 cells could coordinate that signaling with low Cx43 levels via unknown mechanisms. In a normal tissue environment with many G1 cells, Cx43 gap junctions allow for the connectivity of these cells and coordination of long-range Ca^2+^ signaling across the pool of stem cells in different cell cycle phases. These data uncouple long range from local communication and identify Cx43 as a molecular mediator necessary for spatially unrestricted signaling coordination across the basal layer.

### G2-regulated Ca^2+^ signaling feeds back into cell cycle progression

**Figure 5.**
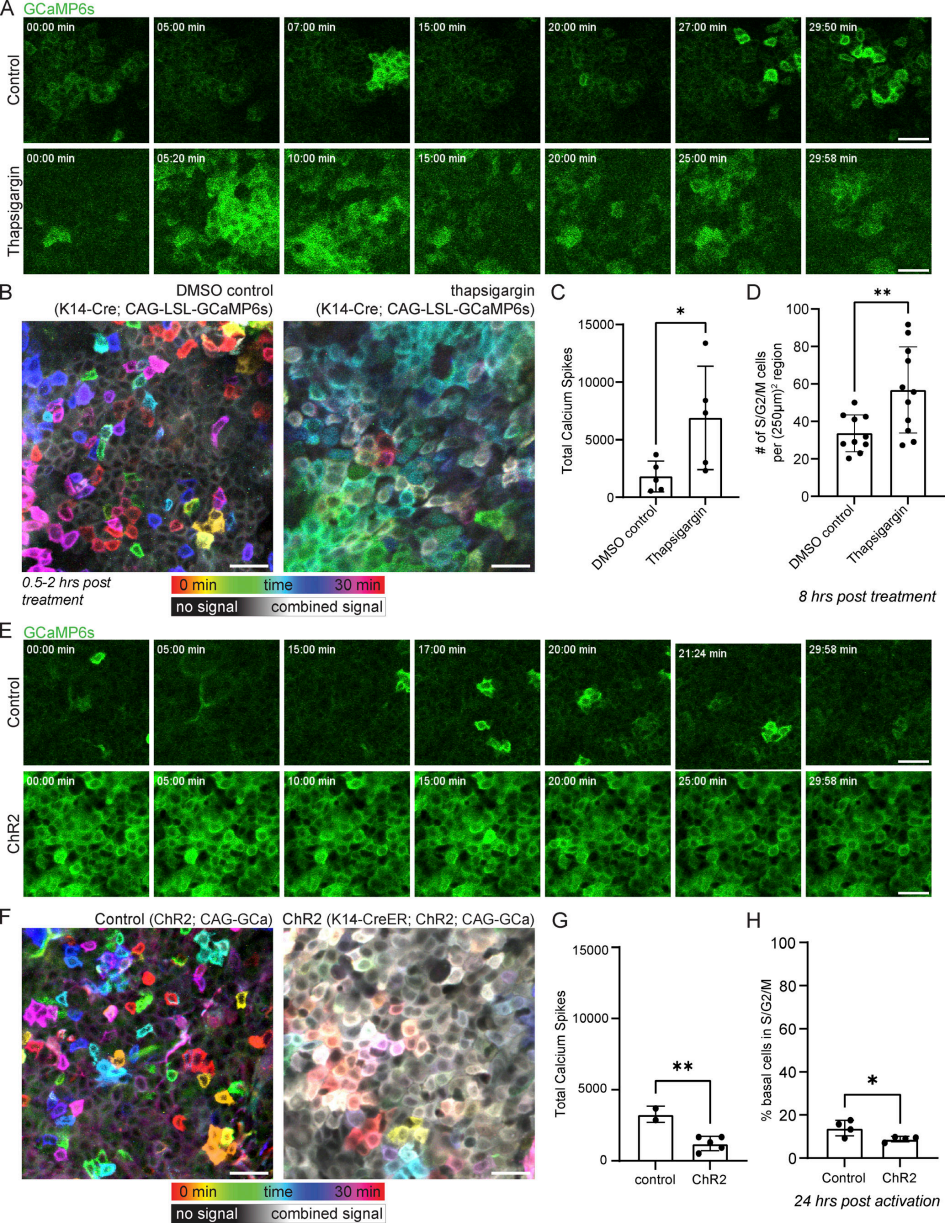
**Coordinated Ca**^**2+**^ **signaling regulates entrance into the cell cycle. (A)** Time-course of signaling from 30-min movies of the stem cell pool of DMSO control- and thapsigargin-treated Ca^2+^-sensor mice 0.5–2 h after treatment. Scale bar: 25 µm. **(B)** Representative max intensity projection of 30-min recordings of the basal layer of DMSO control and thapsigargin treated Ca^2+^-sensor mice, showing increased Ca^2+^ dynamics in the hours after thapsigargin treatment. Color scale indicates GCaMP6s signal across time. Scale bar: 25 µm. **(C)** Total Ca^2+^ events identified in thapsigargin-treated mice versus DMSO treated controls. *P = 0.0417, unpaired two-tailed Student’s *t* test, *n* = 5 control movies and 5 thapsigargin movies. **(D)** Number of cycling S, G2, and M cells in the basal stem cell layer of R26p-Fucci2 mice 8 h after treatment with thapsigargin or DMSO vehicle control. **P = 0.0083, unpaired two-tailed Student’s *t* test, *n* = 6 250 × 250 µm regions from each of the 11 thapsigargin-treated and 10 DMSO vehicle-treated mice. **(E)** Time-course of signaling from 30-min movies of the stem cell pool of control and ChR2 optogenetic Ca^2+^-sensor mice during blue light activation. Scale bar: 25 µm. **(F)** Representative max intensity projection of 30-min recordings of the basal layer of control and ChR2 optogenetic Ca^2+^-sensor mice during light activation, showing increased resting Ca^2+^ levels. Color scale indicates GCaMP6s signal across time. Scale bar: 25 µm. **(G)** Total Ca^2+^ events identified in light-activated ChR2 mice versus genetic controls. **P = 0.0049, unpaired two-tailed Student’s *t* test, *n* = 2 control and 5 ChR2 mice. **(H)** Quantification of epidermal single cell suspensions processed from ears of either *K14CreER; CAG-LSL-ChR2* or control (*CAG-LSL-ChR2*) mice 10 d after induction with 2 mg of tamoxifen and 24 h after exposure to 1 h of constant blue LED light. Cells were gated using a basal marker (anti-CD49f) and stained with Vybrant DyeCycle Violet Stain to determine the cell cycle stage (G1 or S/G2/M). The results show a decrease in basal cells entering S/G2/M in *K14-CreER; CAG-LSL-ChR2* mice compared to controls. *P = 0.0356, unpaired two-tailed Student’s *t* test, *n* = 4 mice per experimental and control groups.

**Figure S9.**
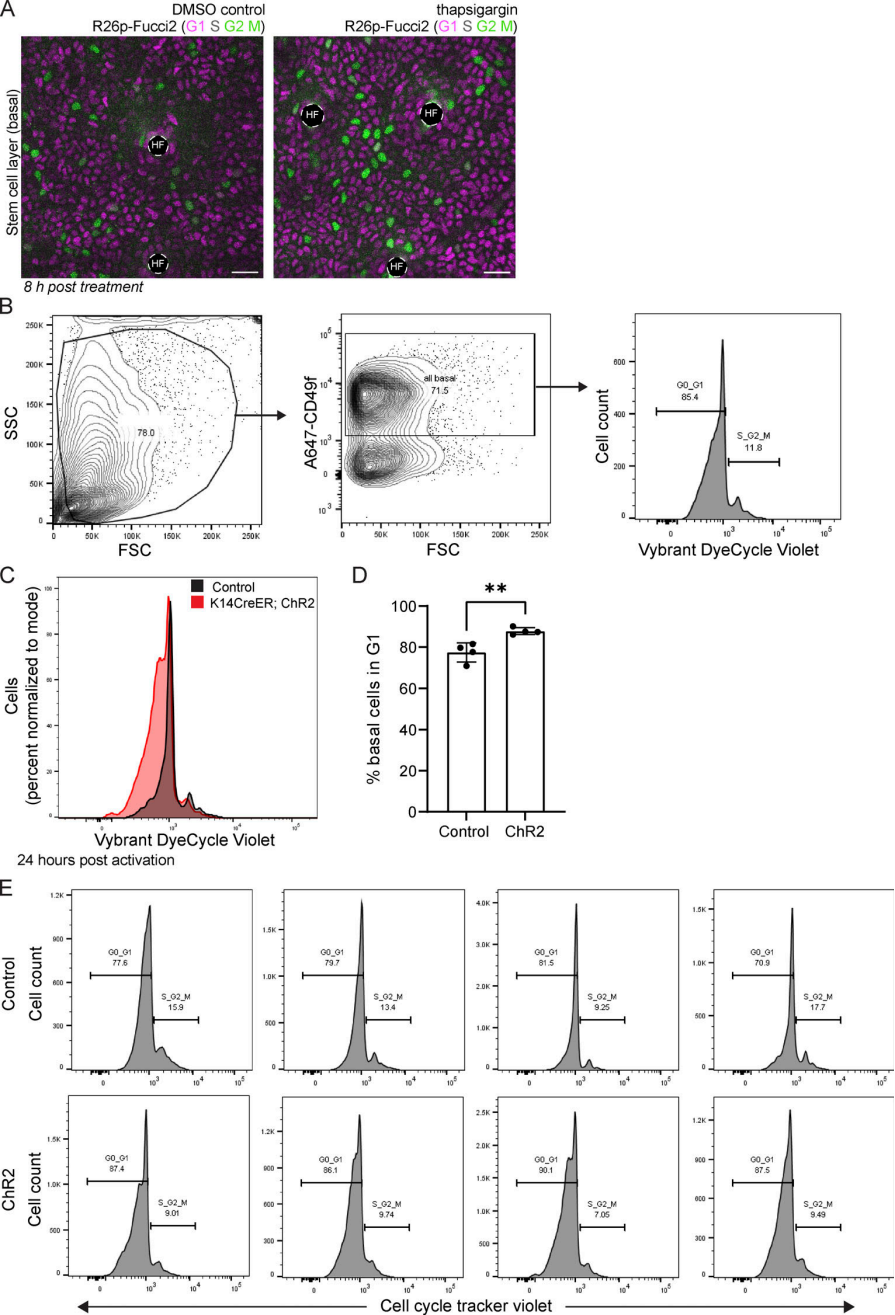
**Changing tissue-wide Ca**^**2+**^ **dynamics affects the cell cycle distribution within the epidermal stem cell layer. (A)** R26p-Fucci2 mice 8 h post-thapsigargin treatment versus DMSO control. G1 cells are in magenta, S cells are double positive for magenta and green and shown in gray, and G2 and M cells are in green. Scale bar: 25 µm. **(B)** Epidermal single cell suspensions were processed from ears of either K14-CreER; ChR2 or control (ChR2) mice 10 d after induction with 2 mg tamoxifen and 24 h after exposure to 1 h of constant blue LED light for flow cytometry and gated for basal cells using anti-CD49f. Cell cycle stage (G1 or S/G2/M) was determined using the gating strategy outlined in the Vybrant DyeCycle Violet Stain product protocol. **(C)** Representative flow cytometry histogram plots of ear basal cells stained with Vybrant DyeCycle Violet comparing K14-CreER; ChR2 (red) and control mice (black) showing a decrease in cells entering S/G2/M in the light-activated K14-CreER;ChR2 mice. **(D)** Quantification of epidermal single cell suspensions processed from ears of either K14-CreER; ChR2 or control (ChR2) mice 10 d after induction with 2 mg of tamoxifen and 24 h after exposure to 1 h of constant blue LED light. Cells were gated using a basal marker (anti-CD49f) and stained with Vybrant DyeCycle Violet Stain to determine cell cycle stage (G1 or S/G2/M). The results show an increase in G1 basal cells in K14-CreER;ChR2 mice compared with controls. **P = 0.0057, unpaired two-tailed Student’s *t* test, *n* = 4 mice per experimental and control groups. **(E)** Replicate flow cytometry histogram plots of ear basal cells stained with Vybrant DyeCycle Violet comparing K14-CreER; ChR2 and control mice showing a decrease in cells entering S/G2/M in the light-activated K14-CreER; ChR2 mice.

Our discoveries so far have broadened our understanding of the organization of stem cell communication via Ca^2+^ and the upstream regulatory principles. We next investigated the role of coordinated Ca^2+^ signaling within the stem cell layer using a combination of drug treatment and optogenetic tools to modify Ca^2+^ dynamics. Based on previous literature showing a role for Ca^2+^ signaling in epithelial regeneration (Gudipaty et al., 2017; Deng et al., 2015), we examined whether there were changes in cell cycle progression after perturbation of Ca^2+^ dynamics. ER Ca^2+^ stores have been shown to be essential for epidermal Ca^2+^ signaling (Foggia et al., 2006; Callewaert et al., 2003; Celli et al., 2011). We first pharmacologically inhibited the activity of the ER-localized pump, sarcoendoplasmic reticulum Ca^2+^ ATPase (SERCA), with topical thapsigargin treatment. Depletion of ER Ca^2+^ stores leads to activation of store-operated calcium entry (SOCE) as the stromal interaction molecule (STIM) senses depletion and opens the Orai Ca^2+^ channel (Berridge, 2016). As a result, we observed a rapid increase in Ca^2+^ dynamics within the basal stem cell layer after thapsigargin treatment (Fig. 5, A–C). We also treated cell cycle reporter mice (*R26p-Fucci2*) with thapsigargin. We quantified the number of green cycling S, G2, and M cells across large regions, finding that thapsigargin treatment increased the number of cycling cells just 8 h after treatment (Fig. 5 D and Fig. S9 A).

**Figure 6.**
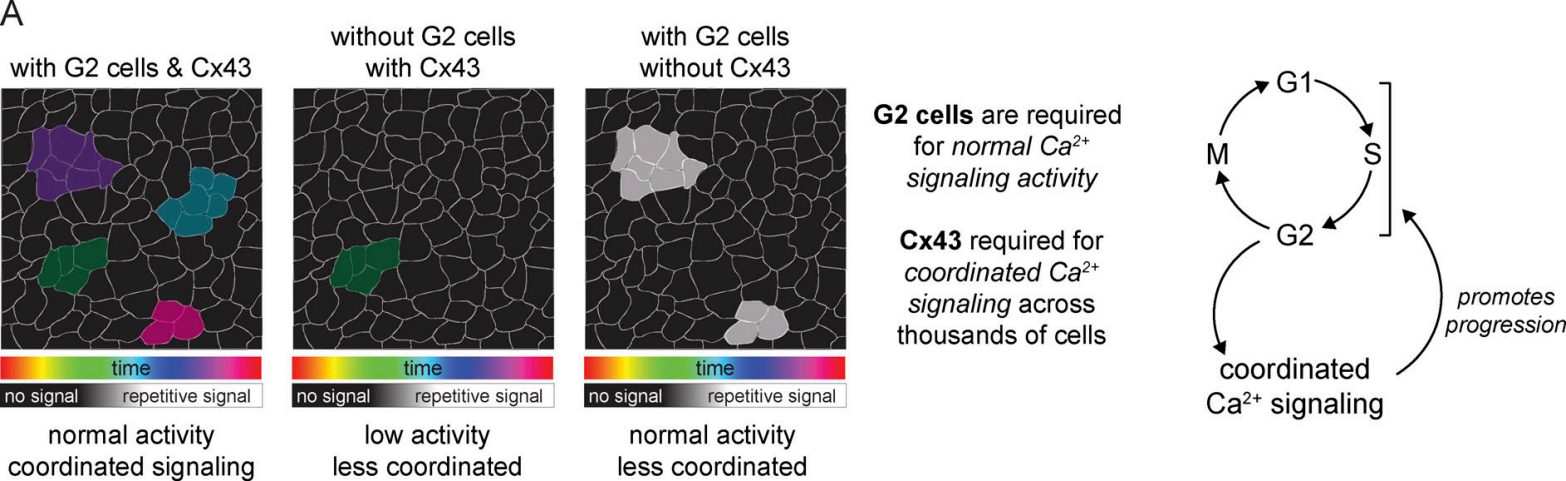
**G2 cells control Ca**^**2+**^ **signaling within the epidermal stem cell layer, which feeds back into cell cycle progression. (A)** Model depicting the cellular and molecular regulators of Ca^2+^ signaling and the role of Ca^2+^ signaling in controlling cell cycle progression.

Conversely, we dampened total Ca^2+^ dynamics by continuously activating optogenetic channelrhodopsin-2 (ChR2) mice (*K14-CreER; CAG-LSL-ChR2*; *CAG-GCaMP6s*) with a blue LED light or the two-photon microscope’s 920 nm Vision laser for 1 h (Fig. 5, E–G). Opposite from when we increased the dynamics of Ca^2+^ signaling, flooding epidermal cells continuously with Ca^2+^ ions via optogenetic activation of the plasma membrane–localized ChR2 ion channel abrogated dynamics and led to a drop in the number of cycling S/G2/M cells detected 24 h later by flow cytometry (Fig. 5 H; and Fig. S9, B–E). These experiments show that Ca^2+^ signaling regulates cell cycle progression within the basal stem cell layer. This may be part of a positive feedback loop, where G2 cells are necessary for the initiation of normal levels of Ca^2+^ signaling, and this Ca^2+^ signaling promotes cell cycle progression in the basal stem cell layer (Fig. 6 A).

## Discussion

As a general principle, regenerative tissues must orchestrate many types of cellular decisions across an ever-changing environment. The role of Ca^2+^ in proliferation, a fundamental property of regenerative tissues, was discovered more than 50 yr ago (Balk, 1971). However, the characteristics and regulation of Ca^2+^ signaling across a complex, multicellular regenerative tissue during homeostasis remains unclear. In this study, we set out to understand how cells orchestrate intercellular Ca^2+^ signaling in the unperturbed epithelial stem cell pool. More broadly, we aimed to understand how regenerative tissues integrate information flow across multiple scales to carry out essential homeostatic behaviors.

Most studies to date have used static analysis, perturbed conditions (such as skin explants), or in vitro models to study Ca^2+^ signaling within the epidermal stem cell pool (Celli et al., 2021; Elias et al., 1998; Tsutsumi et al., 2009; Denda and Denda, 2007). In this study, we developed a unique approach to interrogate and resolve Ca^2+^ dynamics in vivo. By tracking thousands of epidermal stem cells through fast live imaging in the adult mouse skin (Rompolas et al., 2016) in combination with unsupervised machine learning methods to enable the analysis of single-cell Ca^2+^ dynamics, we observed a new unexpected paradigm of homeostatic Ca^2+^ signaling flow that is coordinated and nonrandom across the epidermal stem cell population.

We identified two levels of regulation that coordinate local signaling patterns within the stem cell compartment and enable directed signal flow at long distances. First, we observed that G2 cells are necessary for coordinated signaling activity. Second, we found that neighboring G1 cells use Cx43 gap junctions to connect Ca^2+^ signaling neighborhoods across the whole-stem cell compartment. Finally, we uncovered a functional role for coordinated Ca^2+^ signaling, revealing that it acts as a positive feedback loop for cell cycle progression within the stem cell compartment.

Our comprehensive quantification of local patterns of Ca^2+^ signaling within the basal stem cell compartment reinforced the existence of Ca^2+^ signaling activity within this pool of cells during homeostasis without perturbation (Murata et al., 2018). These findings established that local patterns of Ca^2+^ signaling across neighborhoods of 1–10 cells are prevalent across a pool of heterogeneous stem cells, distinct from larger intercellular Ca^2+^ waves (ICWs) in epithelial sheets that are often a focus of attention in other systems.

Further, we were able to investigate the spread of information across multiple scales by developing GSTH. Importantly, GSTH, a data-driven, unsupervised approach, is highly versatile and can be applied in the future to imaging data with any molecular sensor and across tissues with widely different geometries. GSTH revealed smooth trajectories over time for Ca^2+^ signaling data from the basal stem cell pool, uncovering an emergent property of long-range spatiotemporally coordinated information flow. Our applications of GSTH to explore and understand dynamic cellular signaling open a new realm of possibilities for understanding not only the molecular signaling pathways that underpin regenerative processes in homeostatic and perturbed tissue environments but also any cellular signaling system beyond tissue regeneration.

Our study characterizes the constituent states of the cells within the stem cell layer, finding that stem cells communicate cohesively with their differentiation-committed neighbors, as both populations remain competent to proliferate and respond to tissue demands. Using a new genetic reporter for an early differentiation marker (K10), we show that K10-reporter-positive differentiation-committed cells and K10-reporter-negative stem cells participate equally in Ca^2+^ signaling, although they display subtly different spatiotemporal dynamics. These results demonstrate that the basal layer, as the stem cell compartment, is cohesive in its signaling dynamics and long-range communication.

While Ca^2+^ signaling has been linked to cell proliferation in developmental and regenerative contexts (Gudipaty et al., 2017; Deng et al., 2015), the relationship between cell cycle progression and Ca^2+^ signaling in living animals is poorly understood. We found that when cells are in G2, they display Ca^2+^ signaling patterns that are more similar to each other across spatial and temporal dimensions than when they are in G1 or S. These findings complement recent work in the adult *Drosophila melanogaster* intestine that find fate-specific characteristics of Ca^2+^ dynamics (Kim et al., 2022). Additionally, we found that these G2 cells are essential for tissue-level signaling coordination, in the sense that when the stem cell layer is depleted of G2 cells, smooth signaling dynamics are disrupted and overall signaling activity is lower. We did not uncover the molecular mechanism that G2 cells employ within the tissue to achieve homeostatic levels of Ca^2+^ signaling; however, our analysis of cell cycle–specific transcription of Ca^2+^ signaling pathway genes is a starting point for further investigation.

Our discovery that Cx43, a molecular player implicated in the past as an on/off switch for intercellular Ca^2+^ signaling, is necessary for long-range coordination of Ca^2+^ signaling across the stem cell compartment but not for local clusters of Ca^2+^ transients revealed a complex role for Cx43 gap junctions in this compartment. This contrasts with other work in this context, which has often used drug treatments to target gap junctions and then looked at Ca^2+^ signaling dynamics, showing disruption of all intercellular Ca^2+^ signaling. Therefore, our results build upon recent work (Ho et al., 2021) proposing a more nuanced role of gap junctions in mediating global communication across stem cell pools and suggest that Cx43 plays a unique role in coordinating information flow across this community of stem cells. Importantly, other molecular regulators, including other gap junction proteins such as Cx31, may compensate for the loss of Cx43 to carry out local patterns of Ca^2+^ signaling and allow G2 cells to coordinate Ca^2+^ signaling in the absence of Cx43^high^ G1 cells.

To our knowledge, our study represents the first time that stem cell progression through the cell cycle and Ca^2+^ signaling have been studied in conjunction in vivo. Additionally, while analysis of individual epidermal stem cells gives the impression of localized and random bursts across neighborhoods of 1–10 cells, our global analysis shows an underlying long-range and time-directed coordination of Ca^2+^ signaling across thousands of cells. Together, our results provide insight into how a heterogeneous pool of stem cells carry out different roles to regulate a molecular pathway at a large scale and maintain proper homeostasis, new concepts to the fields of both regenerative biology and Ca^2+^ signaling.

### Limitations of the study

In this study, we increased or decreased intracellular Ca^2+^ dynamics in a global manner across the stem cell compartment to assess the role of coordinated Ca^2+^ signaling at a population level. Further development of optogenetic tools to locally control intracellular Ca^2+^ dynamics will allow for deeper investigation into the relationship between Ca^2+^ signaling and cell cycle progression. Finally, drug treatments can be pleiotropic and the absorption into the skin of different drugs is quite variable. This issue was addressed by using a combination of drug treatments and genetic mouse models throughout the study.

## Materials and methods

### Mice and experimental conditions

*K14-Cre* (Vasioukhin et al., 1999) mice were obtained from E. Fuchs (Rockefeller University, New York, NY). *Rosa26p-Fucci2* (Abe et al., 2013) mice were obtained from S. Aizawa (RIKEN, Tokyo, Japan). *K10-rtTA* (Muroyama and Lechler, 2017) mice were obtained from T. Lechler (Duke University, Durham, NC). *K14-H2B-mCherry* mice were generated in the laboratory and described previously (Mesa et al., 2015). *Cx43^fl/fl^* (Calera et al., 2006; Liao et al., 2001), *Rosa26-CAG-LSL-GCaMP6s* (Madisen et al., 2015), *Rosa26-mTmG* (Muzumdar et al., 2007), *Sox2-Cre* (Hayashi et al., 2002), *K14-CreER* (Vasioukhin et al., 1999), *K14-rtTA* (Xie et al., 1999), *tetO-Cdkn1b* (Pruitt et al., 2013), *Rosa26-CAG-LSL-hChR2(H134R)-tdTomato-WPRE* (Madisen et al., 2012), and *Col1a1-tetO-H2B-mcherry* (Egli et al., 2007) mice were obtained from The Jackson Laboratory. All mice were bred to a CD1 background.

Germline recombined *Rosa26-CAG-GCaMP6s* mice were generated by crossing *Rosa26-CAG-LSL-GCaMP6s* to *Sox2-Cre* mice. To block the cell cycle progression of epithelial cells during G1, *Rosa26-CAG-GCaMP6s* mice were mated with *K14-rtTA*; *tetO-Cdkn1b* mice (*Rosa26-CAG-GCaMP6s; K14-rtTA*; *tetO-Cdkn1b*) and given doxycycline (1 mg ml^−1^) in potable water with 1% sucrose between P45 and P60. Doxycycline treatment was sustained until imaging was performed 3 d later. Siblings without the *tetO-Cdkn1b* allele (*Rosa26-CAG-GCaMP6s; K14-rtTA*) were used as controls. To visualize Ca^2+^ dynamics in *K10*+ differentiation-committed cells, *Rosa26-CAG-GCaMP6s; K10-rtTA*; *Rosa26-Col1a1-tetO-H2B-mCherry* mice were given doxycycline (1 mg ml^−1^) in potable water with 1% sucrose between P45 and P60. Doxycycline treatment was sustained until imaging was performed 4 d later.

Mice from experimental and control groups were randomly selected from either sex for live imaging experiments. No blinding was done. All procedures involving animal subjects were performed under the approval of the Institutional Animal Care and Use Committee (IACUC) of the Yale School of Medicine.

### In vivo imaging

Imaging procedures were adapted from those previously described (Pineda et al., 2015). All imaging was performed in distal regions of the ear skin during prolonged telogen, with hair removed using depilatory cream (Nair) at least 2 d before the start of each experiment (except in the case of drug treatments, when depilation was performed immediately before topical drug application). Mice were anesthetized using an isoflurane chamber and then transferred to the imaging stage and maintained on anesthesia throughout the course of the experiment with vaporized isoflurane delivered by a nose cone (1.25% in oxygen and air). Mice were placed on a 37°C warming pad during imaging. The ear was mounted on a custom-made stage and a glass coverslip was placed directly against it. Image stacks were acquired with a LaVision TriM Scope II (LaVision Biotec) laser scanning microscope equipped with a tunable Two-photon Vision II Ti:Sapphire (Coherent) Ti:Sapphire laser, tunable Two-photon Chameleon Discovery Ti:Sapphire laser (Coherent), and Imspector Pro (LaVision Biotec, v.7.0.129.0). To acquire serial optical sections, a laser beam (940 and 1,120 nm for mice and whole-mount staining) was focused through a 20× or 40× water-immersion lens (NA 1.0 and 1.1, respectively; Zeiss) and scanned with a field of view of 500 or 304 µm^2^, respectively, at 800 Hz or through a 25× water-immersion lens (NA 1.0; Nikon) and scanned with a field of view of 486 µm^2^ at 800 Hz. Z-stacks were acquired in 0.5–3 µm steps to image a total depth of up to 100 µm of tissue. Imaging data were acquired with four Hamamatsu non-descanned detectors, two GaAsP high sensitivity photomultiplier modules (Hamamatsu H7422A-40 and Hamamatsu H7422-40-LV) for green and far-red emission, and photomultiplier modules (Hamamatsu H11901-20 and Hamamatsu H6780-01) for orange and blue emission. Emission colors were separated through LaVision’s spectral detector utilizing a variety of dichroic (T495LPXR, T560LPXR, 620LPXR) and bandpass filters (450/50, 525/50, 610/75, 660/75; Chroma Technology). To visualize large areas, 2–64 tiles of optical fields were imaged using a motorized stage to automatically acquire sequential fields of view. Visualization of collagen was achieved via the second harmonic signal at 940 nm. Various fluorochromes were utilized for each experiment, including AlexaFluor 488, AlexaFluor 568, AlexaFluor 647, EGFP, mCherry, tdTomato, and mVenus. For all timelapse movies, the live mouse remained anesthetized for the length of the experiment, and serial optical sections were captured at intervals of 2 s. For revisits, the same region of live mouse skin was imaged across intervals of multiple days. Anatomical features and patterns of hair follicles and collagen were used as landmarks for finding the same skin location (see Fig. S1 A).

### Image analysis

Raw image stacks were imported into FIJI (ImageJ, National Institutes of Health [NIH]) for analysis. Individual optical planes, summed, or max Z stacks of sequential optical sections were used to assemble figures. To prepare movies where the nuclear signal bleached over the course of the timelapse, we used the Fiji Bleach Correction plugin (Miura, 2020), specifying the Simple Ratio Method. Motion correction of timelapse movies was performed using NoRMCorre (Pnevmatikakis and Giovannucci, 2017).

Segmentation of actively signaling cells was performed using the CaImAn MATLAB package (Giovannucci et al., 2019). In order to segment all cells in the field of view, including inactive cells, we used part of the MATLAB package from Romano et al. (2017), a watershed segmentation method. We normalized the fluorescence intensity of each cell at each timepoint to the minimum fluorescence intensity of that cell as a baseline. From the normalized fluorescence values for each segmented cell, we used peak finding in MATLAB (version R2018b) and then fit Gaussian curves to each peak to be able to quantify spike duration, peak intensity, frequency of flashing, etc. To quantify the neighborhood size of clustered signaling, we created a graph for each timelapse, where each node represented one segmented, spiking cell. We connected nodes that represented cells spiking directly adjacent to one another (spatial neighbors) within 10 s of each other (temporally correlated). We then counted the number of connected nodes to quantify the size of each signaling neighborhood.

To analyze Cx43 immunofluorescence staining in whole-mount tissue from *R26p-Fucci2* mice, epidermal basal cells were hand-segmented using the freehand line function in FIJI with a line width of 10 pixels to account for only the punctate Cx43 signal around each cell. Cx43 fluorescence intensity was measured as the mean fluorescence intensity in each defined circle.

Image correlation coefficients were calculated between two images to compare the similarity between the images (Marsh et al., 2018). Positive image correlation coefficients represent a high overlap of signal, with identical images producing an image correlation coefficient of *r* = 1.0. Negative correlation coefficients mean that the signal in one image correlates with the absence of the signal in the second. Zero correlation is no correlation (50% of the signal overlaps between two images and 50% of the signal does not overlap). A Python script module, run through FIJI, was used to calculate the 2D correlation coefficient between two images.

### Whole-mount staining

Ear tissue was incubated epidermis side up in 5 mg ml^−1^ Dispase II solution (4942078001; Sigma-Aldrich) at 37°C for 15 min and the epidermis was separated from dermis using forceps. The epidermis was fixed in 4% paraformaldehyde in PBS for 15 min at room temperature, washed, and blocked with 0.2% Triton X-100, 5% normal donkey serum, and 1% BSA in PBS. The samples were then incubated with primary antibodies for 12 h at 4°C and with secondary antibodies for ∼2 h at room temperature. Primary antibodies used were as follows: purified mouse anti-Connexin 43, C-terminal, clone P4G9 (1:100; MABT901; Sigma-Aldrich), rabbit anti-Connexin 30.3 polyclonal antibody (1:100; 40-0900; Thermo Fisher Scientific), rabbit anti-Connexin 26 polyclonal antibody (1:100; 71-0500; Thermo Fisher Scientific), rabbit anti-Connexin 31 polyclonal antibody (1:100; 36-5100; Thermo Fisher Scientific), guinea pig anti-K10 (1:200; GP-K10; Progen), and rabbit anti-pH3 (1:300; 06-570; Millipore). All secondary antibodies used were raised in a goat host and were conjugated to AlexaFluor 488, 568, or 647 (Thermo Fisher Scientific). In some cases, tissue was then incubated with Hoechst 33342 (1:500; H3570; Becton Dickinson) for 15 min and then washed with blocking solution. Finally, the tissue was mounted with Vectashield Anti-fade mounting medium (Vector Laboratories) or SlowFade Diamond Antifade Mountant (Thermo Fisher Scientific) and a #1.5 coverslip and imaged on a LaVision TriM Scope II as described in In vivo imaging.

### Tamoxifen induction

To induce the expression of membrane-GFP and/or loss of Cx43 expression, *K14-CreER; Cx43*^*fl/fl*^*; mTmG* mice or *K14-CreER; Cx43*^*fl/fl*^ mice were given three doses of tamoxifen (2 mg in corn oil) 3, 4, and 5 d before imaging or tissue collection by intraperitoneal injection. To observe phenotypes of the total loss of Cx43 just 1 d after recombination, we also topically applied 0.01 mg (Z)-4-hydroxytamoxifen (4-OHT) in an ethanol-vaseline slurry to the ear of *Rosa26-CAG-GCaMP6s; K14-CreER; Cx43*^*fl/fl*^ or *Rosa26-CAG-GCaMP6s; K14-CreER; Cx43*^*+/+*^ mice 1 d before the start of imaging.

### Topical drug treatment

To stall cells as they transition from S to G2 phase of their cell cycles, MMC (Tomasz, 1995) was delivered topically to the ear skin. MMC was dissolved in a 15 mg ml^−1^ stock solution in dimethyl sulfoxide (DMSO) and then diluted 100 times in 100% petroleum jelly (Vaseline; final concentration is 150 mg ml^−1^). 100 μg of the mixture of the MMC and the petroleum jelly was spread evenly on the ear 1 and 2 d before imaging. A mixture of 100% DMSO in petroleum jelly (1:100) was used as vehicle control. Demecolcine was used to block microtubule polymerization (Rieder and Palazzo, 1992). Demecolcine was dissolved in 25 mg ml^−1^ stock solution in DMSO and delivered as described for the MMC treatment.

To block SERCA activity and activate SOCE, mice were treated with thapsigargin or DMSO vehicle control. Thapsigargin was dissolved in a 25 mg ml^−1^ stock solution in DMSO and then diluted 100 times in 100% petroleum jelly (Vaseline; final concentration is 250 mg ml^−1^). 100 μg of the mixture of thapsigargin and petroleum jelly was spread evenly on the ear of the anesthetized mouse 30 min (Ca^2+^ imaging experiments) or 8 h (cell cycle experiments) before imaging. A mixture of 100% DMSO in petroleum jelly (1:100) was used as vehicle control.

### Transmission electron microscopy

Ear skin tissue from *K14-CreER; Cx43*^*fl/fl*^ mice 5 d after tamoxifen induction and matching controls were fixed in a buffer. Samples were postfixed with 1% osmium tetroxide, dehydrated through a graded series of ethanol, and embedded in Epon (Electron Microscopy Sciences). Thin sections (70 nm) were cut on a Leica EM UC7 and collected on formvar-coated slot grids (Electron Microscopy Sciences). Sections were stained with 5% Uranyl acetate in 50:50 methanol:water, rinsed, and then stained with lead citrate, ready to use (Electron Microscopy Sciences). They were imaged with a Hitachi H-7650 transmission EM at 80 kV.

### Analysis of single-cell RNA sequencing data

To compare gene expression of Ca^2+^ signaling pathway genes between different cell cycle stages, we used a previously published integrated single-cell RNA-sequencing dataset consisting of interfollicular epidermal cells from mouse dorsal skin (Cockburn et al., 2022). In brief, this dataset includes annotations for basal and suprabasal cell populations (based on ITGA6 sorting) and cell-cycle stages based on a previously published list of cell cycle markers (Tirosh et al., 2016). For subsequent analysis, only basal cells were included. Using Scanpy (Wolf et al., 2018), log-normalized counts of Ca^2+^ signaling pathway genes were grouped by cell cycle stage to generate gene expression heatmaps. We used the Kyoto Encyclopedia of Genes and Genomes (KEGG) annotation database’s Ca^2+^ signaling pathway genes, plus some select genes from the literature, to create our Ca^2+^ signaling pathway gene list.

### Optogenetic activation

To activate Channelrhodopsin-2 (ChR2) (*K14-CreER; Rosa26-CAG-LSL-ChR2*; *n* = 4) mice for the cell cycle experiment, we anesthetized them along with littermate controls (*Rosa26-CAG-LSL-ChR2*; *n* = 4) with 100 μl per 12 g of body weight of rodent cocktail (15 mg ml^−1^ ketamine; 1 mg ml^−1^ Xylazine) 10 d after receiving three intraperitoneal injections of tamoxifen (2 mg in corn oil per day for 3 consecutive days). We then placed them in a cage with a strip of blue LED lights 1 cm from their ears for 60 min. To activate ChR2 (*K14-CreER; Rosa26-CAG-LSL-ChR2*; *Rosa26-CAG-GCaMP6s*) mice for the Ca^2+^ imaging experiment, we injected them along with littermate controls (*Rosa26-CAG-LSL-ChR2*; *Rosa26-CAG-GCaMP6s*) with three intraperitoneal injections of tamoxifen (2 mg in corn oil per day) for 3 consecutive days. A week later, we simultaneously imaged and activated these mice with the two-photon microscope’s 920 nm Vision laser.

### Flow cytometry

Epidermal single-cell suspensions were prepared from ears of ChR2 (*K14-CreER; Rosa26-CAG-LSL-ChR2*; *n* = 4) mice and littermate controls (*Rosa26-CAG-LSL-ChR2*; *n* = 4) 24 h after exposure to blue light for flow cytometry with a protocol adapted from previously described methods (Mohammed et al., 2016). Briefly, single-cell suspensions of epidermal cells were obtained from ear skin and incubated for 30 min at 37°C in 0.3% trypsin (Sigma-Aldrich) in 150 mM NaCl, 0.5 mM KCl, and 0.5 mM glucose. The epidermis was separated from the dermis with tweezers, minced, and the resulting cells were crushed and filtered through a 70 µm filter. All samples were pretreated with rat serum (Sigma-Aldrich) and incubated with anti-CD49f (1/100; 313610; Biolegend) for 30 min at 4°C. The samples were then washed with Hanks’ balanced salt solution (HBSS; 14170-112; Gibco) centrifuged at 300 *g* for 5 min at 4°C and resuspended in Vybrant DyeCycle Violet Stain (V35003; Invitrogen) at a final concentration of 2.5 µM in HBSS. The cells were stained for 30 min protected from light at 37°C and then immediately run on a Becton Dickinson LSRII outfitted with Diva software v8.0.1, and the data were analyzed using Flowjo v10.6.2.b.

### Statistics and reproducibility

Statistical analyses were performed using GraphPad Prism (version 9.2) software (GraphPad, Inc.). Statistical parameters are reported in figure legends. Statistical comparisons were made using an unpaired two-tailed Student’s *t* test, Mann–Whitney test, or the one-way analysis of variance (ANOVA) with multiple comparison’s test. Differences between the groups were considered significant at P < 0.05 and the data are presented as means ± SD, except for the violin plots, which show the median and quartiles as dashed lines. Data distribution was assumed to be normal, but this was not formally tested.

### GSTH

Consider the problem of representing a signal on a set of cells, e.g., epidermal stem cells arranged in planar spatial patterns. If we simply describe the signals as a vector of values indexed by cells in some order, then we would not be able to compare signaling patterns from different tissues, as specific cellular coordinates are not matched between tissues. Therefore, the signaling description has to be invariant to permutations in cell indexing, shifts in the signal, and even differences in the number of cells. To address this issue, in classic signal processing, researchers use frequency domain descriptions, such as the Fourier transform (FT), which describes the periodicity rather than the time- or space-specificity of signals. With the prevalence of graph-structured data, there is an emerging field of graph signal processing (Shuman et al., 2013), in which researchers have invented the analogous graph Fourier transform (GFT; Sandryhaila and Moura, 2013). In our case, the graph consists of cells as vertices, and edges are determined by physical adjacency. However, the GFT (and the FT) is usually only suitable for describing signals with global periodic patterns. More localized signaling patterns can be described using wavelet transforms. We developed GSTH (Bhaskar et al., 2023 *Preprint*), based on the graph wavelet transform (Coifman and Maggioni, 2006), to capture both localized and diffuse signaling patterns across the cellular graph. It consists of four steps (Fig. 1 K, top), which are explained below:

#### Step 1

The first step of GSTH consists of defining a graph on which the Ca^2+^ is regarded as a static node signal for each timepoint. For the epithelial cells, we defined a nearest-neighbors graph *G* = {*V*,*E*} with each vertex *v*_*i*_ ϵ *V* being a cell, and an edge (*v*_*i*_, *v*_*j*_) ϵ *E* if the cells *v*_*i*_ and *v*_*j*_ are spatially adjacent. The connectivity of this cell graph *G* can be described by its adjacency matrix *A*, where *A*_*ij*_ = 1 if *v*_*i*_ and *v*_*j*_ are connected (i.e., (*v*_*i*_, *v*_*j*_) ϵ *E*) and 0 otherwise (Fig. 1 K, step 1). The vertex degrees are collected in a degree matrix *D*, with $D_{ii}=\sum_{j=1}^{i}A_{ij}\text{.}$ The Ca^2+^ signaling level of the cell is regarded as a signal *x* defined on the graph, i.e., each vertex *v*_*i*_ in the graph has an activation value *x*(*v*_*i*_, *t*) representing the level of Ca^2+^ signaling at time *t*. From here on, this graph is referred to as the cellular graph.

#### Step 2

The second step is to derive a mathematical descriptor of the cellular signaling pattern defined in step 1. We utilize the geometric scattering transform (Fig. 1 K, step 2a), which is a generalization of scattering transforms to graphs, to embed each timepoint. The geometric scattering transform consists of a cascade of graph wavelet transforms followed by a nonlinear modulus operation applied to graph signals and is built in a multilayer (or multiorder) architecture, each extracting finer descriptions of signals. Graph wavelets, in turn, are defined using a diffusion operator (or equivalently a random walk operator) on the graph and are essentially a difference between signal diffusions of different time steps on the graph. Thus, we first define a diffusion operator $R=\frac{1}{2}\left(I+AD^{-1}\right),$ where *A* and *D* are the graph adjacency matrix and degree matrix, respectively, from step 1 and *I* is the identity matrix. This diffusion operator computes the probability of a lazy random walk, starting from any vertex and ending in another vertex in *t* steps, where each step (from one vertex to another) has a probability proportional to its adjacency and inversely to the degree. Hence, the *t*-th power of *R*, *R*^*t*^, represents the probability distribution after *t* steps. Based on this operator *R*, we can then define a graph wavelet of different scales as follows (Coifman and Maggioni, 2006):

$$\Psi_{0}=I-R\;,\;\Psi_{j}=R^{2^{j-1}}-R^{2^{j}}=R^{2^{j-1}}(I-R^{2^{j-1}})\;,\;j\geq 1\;.$$ (1)

By using multiple wavelet scales Ψ*_j_*_1_,Ψ*_j_*_2_,Ψ*_j_*_3…_, the operators can thus be used to extract multilevel signal information from the graph. We then calculate the scattering transform *S*(x) at different orders, which yields a collection of scattering coefficients *S*(x(*t*)) as overall cellular signaling pattern embeddings for timepoint *t*. Specifically, the zeroth-order scattering transform calculates the local averaging of raw signals x(*t*_*i*_) at vertex/cell *v*_l_ and is obtained by:

$$S_{0}(x(v_{l},t_{i}))=R^{2^{J}}x(v_{l},t_{i}).$$ (2)

The resulting scattering coefficients $S_{0}(x(v_{l},t_{i}))$ for every vertex/cell at timepoint *t*_*i*_ are then concatenated to form the zeroth-order scattering coefficients $S_{0}(x(t_{i}))$ for timepoint *t*_*i*_*.* While the diffusion operator *R* provides local averaging of neighboring cell patterns, it also suppresses high-frequency information. We can further calculate the first-order scattering coefficients to retrieve fine-grained descriptions of high-frequency information. This is achieved by applying graph wavelets described above:

$$S_{1}(x(j,v_{l},t_{i}))=R^{2^{J}}|\Psi_{j}x(v_{l},t_{i})|,\;1\leq j\leq J,$$ (3)

Similarly, the zeroth-order and first-order scattering coefficients can be further complemented by the second-order scattering coefficients:

$$S_{2}(x(j,j',v_{l},t_{i}))=R^{2^{J}}|\Psi_{j'}|\Psi_{j}x(v_{l},t_{i})||,1\leq j<j'\leq J$$ (4)

Finally, we concatenate the zeroth-order, first-order, and second-order scattering coefficients to form the timepoint embedding $S(x(t_{i}))$ for timepoint *t*_*i*_.

#### Step 3

In the third step, we reduce the descriptors of signaling patterns of each timepoint *S*(*X*(*t*)) from step 2 into a low-dimensional trajectory *E*, where $E=\left\{e_{t1},e_{t2},\ldots ,e_{tn}\right\}$ with each *e*_*i*_ corresponding to a low-dimensional PHATE embedding of timepoint *t*_*i*_*.* This is achieved by applying PHATE, a dimensionality reduction method that captures both local and global nonlinear structures by constructing a diffusion geometry (Moon et al., 2019). The advantage of PHATE over other methods is that it retains trajectory structure and global distances as opposed to stochastic neighbor embeddings, such as UMAP and t-SNE, which tend to shatter trajectory structure. Once we apply PHATE, we can reduce the high-dimensional scattering coefficients *S*(*X*(*t*)) for each timepoint into the 3D PHATE embedding coordinates $E(t)=(E_{1}(x_{t}),E_{2}(x_{t}),E_{3}(x_{t}))$ The collection of these timepoints forms a low-dimensional trajectory $E=\left\{E\left(t_{1}\right),E\left(t_{2}\right)\ldots ,E\left(t_{n}\right)\right\},$ allowing visualization of the temporal dynamics of cells signaling (Fig. 1 K, step 3a).

#### Step 4

Finally, to quantify features of the trajectories *E* obtained in step 3 (e.g., to compare the existence of loops or holes in the trajectories), we quantify their shape using persistent homology, a topological data analysis method (Fig. 1 K, step 4a). In persistent homology calculations, data is gradually coarse-grained by merging nearby points, and at each level of granularity, a graph (or simplicial complex in higher dimensions) of the data is quantified by counting the number of connected components, cycles, and potentially higher-dimensional “holes” (Carlsson, 2009). The gradual coarse-graining is referred to as “filtration.” Here, we use a filtration method known as the Vietoris-Rips filtration, where we create connections between two points *e*_*i*_ and *e*_*j*_ in the trajectory if the points are closer than a threshold *ϵ*, measured using the Euclidean distance on the embedding. The threshold *ϵ* is gradually increased, ranging from 0 to ∞, until all points are connected in a fully connected graph.

We visualize the results in two ways: first, we calculate a persistence diagram *Q* that tracks the birth and death of each topological feature that occurs during the filtration process, i.e., it contains a point at (*ϵ*_*i*_, *ϵ*_*j*_) for each connected component (for instance) that is created at threshold ϵ_*i*_ and destroyed (i.e., merged) at threshold ϵ_*j*_*.* Persistent homology is still under-explored in data science, but it is a robust tool for analyzing the shape features of datasets across multiple scales with guaranteed stability properties, i.e., ensuring small changes in the input data lead to small changes in the homological features (Cohen-Steiner et al., 2007). For instance, we can differentiate between smoother trajectories (which will only contain large cycles appearing at later granularities, i.e., higher values of ϵ) versus rougher trajectories (where cycles can get created and destroyed at smaller values of *ϵ*). Moreover, dynamic trajectories from different mice can be readily compared using well-defined distance metrics between the corresponding persistence diagrams. Here, we use the 2-Wasserstein distance (Cohen-Steiner et al., 2010) to quantitatively compare persistence diagrams obtained from PHATE trajectories. The Wasserstein distance between persistence diagrams *Q*_1_ and *Q*_2_ measures the minimum cost required to transport points in one diagram to the other:

$$W_{2}\left(Q_{1},Q_{2}\right)=\underset{m:Q_{1}\to Q_{2}}{\inf}{\left(\sum_{q_{1}\in Q_{1}}∥q_{1}-m\left(q_{1}\right){∥}^{m}\right)}^{\frac{1}{m}}.$$ (5)

The transportation cost is measured by the Euclidean distance between matching pairs of points, and the overall cost is minimized by finding the best matching function. In other words, Wasserstein distance measures the minimum amount of energy required to transform one persistence diagram into the other.

A second way to visualize the results is through the so-called Betti curves that are associated with a persistence diagram *Q*, resulting in a simple summary curve *B*(*Q*,*q*) for the persistence diagram in dimension *q*. More precisely, the Betti curve of dimension *q* of a diagram *Q* refers to the sequence of Betti numbers, i.e., active topological features, of dimension *q* in *Q*, evaluated for each threshold ϵ. It is a useful descriptor for numerous machine-learning tasks (Rieck et al., 2020). Intuitively, the Betti numbers represent the number of *q*-dimensional holes in a topological space. Therefore, the Betti curve characterizes the connectivity of VR_*s*_(*E*) and, by extension, of the Ca^2+^ signaling data.

### GSTH-based cell embeddings

In addition to the time trajectory, we also create embeddings of the cells (henceforth referred to as the cell embeddings), based on all points in time, to compare the Ca^2+^ signaling patterns of different individual cells within the same experiment (Fig. 1 K, bottom). We utilize the same cellular graph as described in step 1 of the GSTH methodology, and we use the same diffusion operator $R=\frac{1}{2}(I+AD^{-1})$, where *A* and *D* are the graph adjacency matrix and degree matrix, respectively. Using this operator, we define graph wavelets Ψ like in GSTH step 2:

$$\Psi_{j}=R^{2^{j-1}}-R^{2^{j}}$$ (6)

However, unlike before when we considered the signals from all the cells at each individual timepoint, we now consider the signals $x(v_{i},t)=\{x(v_{i},t_{1}),x(v_{i},t_{2}),\ldots ,x(v_{i},t_{T})\}$, which are defined for each vertex/cell *v*_*i*_ across all timepoints as features (Fig. 1 K, step 2b). In this setting, the scattering transform aggregates signals across time:

$$S_{0}(x(v_{i},t))=R^{2^{J}}x(v_{i},t),$$

$$S_{1}(x(j,v_{i},t))=R^{2^{J}}|\Psi_{j}x(v_{i},t)|,\;1\leq j\leq J,$$

$$S_{2}(x(j,j',v_{i},t))=R^{2^{J}}|\Psi_{j'}|\Psi_{j}x(v_{i},t)||,\;1\leq j<j'\leq J.$$ (7)

These wavelet coefficients represent the patterns from the cell itself and incorporate larger-scale signaling patterns by considering neighboring cells at multiple scales. By concatenating these wavelet coefficients at every timepoint for each cell, we compute an embedding for each cell that encodes Ca^2+^ signaling information from that cell and its neighbors across all timepoints. Therefore, the embeddings reflect the Ca^2+^ signaling pattern of an individual cell across all timepoints. Like GSTH, we use PHATE to generate low-dimensional visualizations of the cell embeddings. In the embedding space, cells that have similar signaling patterns are clustered together (Fig. 1 K, step 3b). In heterogeneous populations where cells from multiple experimental conditions are present, we compute a relative likelihood estimate of observing each cell in each of the experimental conditions using MELD (Burkhardt et al., 2021). MELD models the embedding space as a smooth low-dimensional manifold. It calculates a cell density estimate which quantifies the density of each cell over the manifold of all possible experimental conditions. Finally, the difference in density estimates for each cell is used to calculate a relative likelihood, which quantifies the effect of an experimental perturbation as the likelihood of observing each cell in each experimental condition (Fig. 1 K, step 4b).

### Online supplemental material

Fig. S1 shows the dynamic nature of Ca^2+^ signaling in the basal stem cell layer across brief time periods (30 min) and revisits (hours–days). Fig. S2 shows two ways in which K10+ and K10− cells have similar spatiotemporal signaling characteristics. Fig. S3 details how GSTH analysis of Ca^2+^ signaling patterns in the basal stem cell layer reveals coordination and directed spread of signals across time. Fig. S4 shows cell cycle–specific Ca^2+^ signaling. Fig. S5 shows how enrichment of G2 cells does not disrupt coordinated Ca^2+^ signaling. Fig. S6 shows scRNAseq analysis. Fig. S7 shows that loss of Cx43 does not disrupt all gap junctions in the basal stem cell layer. Fig. S8 shows the GSTH analysis of Cx43 cKO. Fig. S9 shows disruption of cell cycle distribution within the basal stem cell layer after increasing or dampening Ca^2+^ signaling. Video 1 shows the long-range coordination of clusters of Ca^2+^ signaling across the basal stem cell layer. Video 2 shows that mitotic cells do not participate in homeostatic Ca^2+^ signaling. Video 3 shows that basal cells stalled in G1 of their cell cycle show low levels of Ca^2+^ signaling. Video 4 is an example of basal cells stalled in mitosis showing low levels of Ca^2+^ signaling. Video 5 shows that basal cells stalled in G2 of their cell cycle show normal patterns of Ca^2+^ signaling. Video 6 shows that Cx43 orchestrates Ca^2+^ signaling at large scales, but not across local neighborhoods, in the stem cell pool. Table S1 is a list of all the genes analyzed in the scRNAseq dataset.

## Supplementary Material

Table S1 lists all the genes analyzed in the scRNAseq dataset.

## Data Availability

All data from this study are available from the corresponding author upon reasonable request. The MATLAB scripts for the image analysis can be downloaded from https://github.com/jesslmoore/CaPeaks. The source code for GSTH and the cell embeddings can be downloaded from https://github.com/krishnaswamylab/GSTH.

## References

[bib1] Abe, T., A. Sakaue-Sawano, H. Kiyonari, G. Shioi, K. Inoue, T. Horiuchi, K. Nakao, A. Miyawaki, S. Aizawa, and T. Fujimori. 2013. Visualization of cell cycle in mouse embryos with Fucci2 reporter directed by Rosa26 promoter. Development. 140:237–246. 10.1242/dev.084111 23175634

[bib2] Balaji, R., C. Bielmeier, H. Harz, J. Bates, C. Stadler, A. Hildebrand, and A.-K. Classen. 2017. Calcium spikes, waves and oscillations in a large, patterned epithelial tissue. Sci. Rep. 7:42786. 10.1038/srep42786 28218282 PMC5317010

[bib3] Balk, S.D. 1971. Calcium as a regulator of the proliferation of normal, but not of transformed, chicken fibroblasts in a plasma-containing medium. Proc. Natl. Acad. Sci. USA. 68:271–275. 10.1073/pnas.68.2.271 5277067 PMC388915

[bib4] Behne, M.J., S. Sanchez, N.P. Barry, N. Kirschner, W. Meyer, T.M. Mauro, I. Moll, and E. Gratton. 2011. Major translocation of calcium upon epidermal barrier insult: Imaging and quantification via FLIM/fourier vector analysis. Arch. Dermatol. Res. 303:103–115. 10.1007/s00403-010-1113-9 21193994 PMC4548958

[bib5] Berridge, M.J. 2016. The inositol trisphosphate/calcium signaling pathway in health and disease. Physiol. Rev. 96:1261–1296. 10.1152/physrev.00006.2016 27512009

[bib6] Bhaskar, D., J.L. Moore, F. Gao, B. Rieck, F. Khasawneh, E. Munch, V. Greco, and S. Krishnaswamy. 2023. Capturing spatiotemporal signaling patterns in cellular data with geometric scattering trajectory homology. bioRxiv. (Preprint posted March 22, 2023). 10.1101/2023.03.22.533807

[bib7] Braun, K.M., C. Niemann, U.B. Jensen, J.P. Sundberg, V. Silva-Vargas, and F.M. Watt. 2003. Manipulation of stem cell proliferation and lineage commitment: Visualisation of label-retaining cells in wholemounts of mouse epidermis. Development. 130.21:5241–5255. 10.1242/dev.00703 12954714

[bib8] Burkhardt, D.B., J.S. Stanley III, A. Tong, A.L. Perdigoto, S.A. Gigante, K.C. Herold, G. Wolf, A.J. Giraldez, D. van Dijk, and S. Krishnaswamy. 2021. Quantifying the effect of experimental perturbations at single-cell resolution. Nat. Biotechnol. 39:619–629. 10.1038/s41587-020-00803-5 33558698 PMC8122059

[bib9] Calera, M.R., H.L. Topley, Y. Liao, B.R. Duling, D.L. Paul, and D.A. Goodenough. 2006. Connexin43 is required for production of the aqueous humor in the murine eye. J. Cell Sci. 119:4510–4519. 10.1242/jcs.03202 17046998

[bib10] Callewaert, G., J.B. Parys, H. De Smedt, L. Raeymaekers, F. Wuytack, J. Vanoevelen, K. Van Baelen, A. Simoni, R. Rizzuto, and L. Missiaen. 2003. Similar Ca^2+^-signaling properties in keratinocytes and in COS-1 cells overexpressing the secretory-pathway Ca^2+^-ATPase SPCA1. Cell Calcium. 34:157–162. 10.1016/S0143-4160(03)00070-8 12810057

[bib86] Carlsson, G. 2009. Topology and data. Bull. Am. Math. Soc. 46:255–308. 10.1090/S0273-0979-09-01249-X

[bib11] Celli, A., C.L. Tu, E. Lee, D.D. Bikle, and T.M. Mauro. 2021. Decreased calcium-sensing receptor expression controls calcium signaling and cell-to-cell adhesion defects in aged skin. J. Invest. Dermatol. 141:2577–2586. 10.1016/j.jid.2021.03.025 33862069 PMC8526647

[bib12] Celli, A., D.S. Mackenzie, D.S. Crumrine, C.L. Tu, M. Hupe, D.D. Bikle, P.M. Elias, and T.M. Mauro. 2011. Endoplasmic reticulum Ca^2+^ depletion activates XBP1 and controls terminal differentiation in keratinocytes and epidermis. Br. J. Dermatol. 164.1:16–25. 10.1111/j.1365-2133.2010.10046.x 20846312 PMC3010253

[bib13] Celli, A., D.S. Mackenzie, Y. Zhai, C.-L. Tu, D.D. Bikle, W.M. Holleran, Y. Uchida, and T.M. Mauro. 2012. SERCA2-controlled Ca²^+^-dependent keratinocyte adhesion and differentiation is mediated via the sphingolipid pathway: A therapeutic target for Darier’s disease. J. Invest. Dermatol. 132:1188–1195. 10.1038/jid.2011.447 22277942 PMC3305850

[bib14] Chen, T.-W., T.J. Wardill, Y. Sun, S.R. Pulver, S.L. Renninger, A. Baohan, E.R. Schreiter, R.A. Kerr, M.B. Orger, V. Jayaraman, . 2013. Ultrasensitive fluorescent proteins for imaging neuronal activity. Nature. 499:295–300. 10.1038/nature12354 23868258 PMC3777791

[bib83] Churko, J.M., and D.W. Laird. 2011. Gap Junctions. *In* Cellular Domains. I.R. Nabi, editor. Wiley Online Library. pp. 339–347. Available at: 10.1002/9781118015759.ch20.

[bib16] Cockburn, K., K. Annusver, D.G. Gonzalez, S. Ganesan, D.P. May, K.R. Mesa, K. Kawaguchi, M. Kasper, and V. Greco. 2022. Gradual differentiation uncoupled from cell cycle exit generates heterogeneity in the epidermal stem cell layer. Nat. Cell Biol. 24:1692–1700. 10.1038/s41556-022-01021-8 36357619 PMC9729105

[bib17] Cohen-Steiner, D., H. Edelsbrunner, J. Harer, and Y. Mileyko. 2010. Lipschitz functions have Lp-stable persistence. Found. Comput. Math. 10:127–139. 10.1007/s10208-010-9060-6

[bib87] Cohen-Steiner, D., H. Edelsbrunner, and J. Harer. 2007. Stability of persistence diagrams. Discrete Comput. Geom. 37:103–120. 10.1007/s00454-006-1276-5

[bib18] Coifman, R.R., and M. Maggioni. 2006. Diffusion wavelets. Appl. Comput. Harmon. Anal. 21:53–94. 10.1016/j.acha.2006.04.004

[bib19] Darbellay, B., L. Barnes, W.H. Boehncke, J.H. Saurat, and G. Kaya. 2014. Reversal of murine epidermal atrophy by topical modulation of calcium signaling. J. Invest. Dermatol. 134:1599–1608. 10.1038/jid.2013.524 24317393

[bib20] Denda, M., and S. Denda. 2007. Air-exposed keratinocytes exhibited intracellular calcium oscillation. Skin Res. Technol. 13:195–201. 10.1111/j.1600-0846.2007.00210.x 17374062

[bib21] Deng, H., A.A. Gerencser, and H. Jasper. 2015. Signal integration by Ca^2+^ regulates intestinal stem-cell activity. Nature. 528:212–217. 10.1038/nature16170 26633624 PMC4669953

[bib22] Dolmetsch, R.E., R.S. Lewis, C.C. Goodnow, and J.I. Healy. 1997. Differential activation of transcription factors induced by Ca^2+^ response amplitude and duration. Nature. 386:855–858. 10.1038/386855a0 9126747

[bib23] Dolmetsch, R.E., K. Xu, and R.S. Lewis. 1998. Calcium oscillations increase the efficiency and specificity of gene expression. Nature. 392:933–936. 10.1038/31960 9582075

[bib24] Egli, D., J. Rosains, G. Birkhoff, and K. Eggan. 2007. Developmental reprogramming after chromosome transfer into mitotic mouse zygotes. Nature. 447:679–685. 10.1038/nature05879 17554301

[bib25] Elias, P.M., S.K. Ahn, M. Denda, B.E. Brown, D. Crumrine, L.K. Kimutai, L. Kömüves, S.H. Lee, and K.R. Feingold. 2002. Modulations in epidermal calcium regulate the expression of differentiation-specific markers. J. Invest. Dermatol. 119:1128–1136. 10.1046/j.1523-1747.2002.19512.x 12445203

[bib26] Elias, P.M., P. Nau, K. Hanley, C. Cullander, D. Crumrine, G. Bench, E. Sideras-Haddad, T. Mauro, M.L. Williams, and K.R. Feingold. 1998. Formation of the epidermal calcium gradient coincides with key milestones of barrier ontogenesis in the rodent. J. Invest. Dermatol. 110:399–404. 10.1046/j.1523-1747.1998.00151.x 9540982

[bib27] Foggia, L., I. Aronchik, K. Aberg, B. Brown, A. Hovnanian, and T.M. Mauro. 2006. Activity of the hSPCA1 Golgi Ca^2+^ pump is essential for Ca^2+^-mediated Ca^2+^ response and cell viability in Darier disease. J. Cell Sci. 119:671–679. 10.1242/jcs.02781 16467572

[bib28] Giovannucci, A., J. Friedrich, P. Gunn, J. Kalfon, B.L. Brown, S.A. Koay, J. Taxidis, F. Najafi, J.L. Gauthier, P. Zhou, . 2019. CaImAn an open source tool for scalable calcium imaging data analysis. eLife. 8:e38173. 10.7554/eLife.38173 30652683 PMC6342523

[bib29] Gudipaty, S.A., J. Lindblom, P.D. Loftus, M.J. Redd, K. Edes, C.F. Davey, V. Krishnegowda, and J. Rosenblatt. 2017. Mechanical stretch triggers rapid epithelial cell division through Piezo1. Nature. 543:118–121. 10.1038/nature21407 28199303 PMC5334365

[bib30] Hayashi, S., P. Lewis, L. Pevny, and A.P. McMahon. 2002. Efficient gene modulation in mouse epiblast using a Sox2Cre transgenic mouse strain. Mech. Dev. 119:S97–S101. 10.1016/S0925-4773(03)00099-6 14516668

[bib31] Hennings, H., D. Michael, C. Cheng, P. Steinert, K. Holbrook, and S.H. Yuspa. 1980. Calcium regulation of growth and differentiation of mouse epidermal cells in culture. Cell. 19:245–254. 10.1016/0092-8674(80)90406-7 6153576

[bib32] Hiratsuka, T., Y. Fujita, H. Naoki, K. Aoki, Y. Kamioka, and M. Matsuda. 2015. Intercellular propagation of extracellular signal-regulated kinase activation revealed by in vivo imaging of mouse skin. eLife. 4:e05178. 10.7554/eLife.05178 25668746 PMC4337632

[bib88] Ho, K.Y.L., R.J. Khadilkar, R.L. Carr, and G. Tanentzapf. 2021. A gap-junction-mediated, calcium-signaling network controls blood progenitor fate decisions in hematopoiesis. Curr. Biol. 31:4697–4712. 10.1016/j.cub.2021.08.027 34480855

[bib33] Isakson, B.E., W.H. Evans, and S. Boitano. 2001. Intercellular Ca^2+^ signaling in alveolar epithelial cells through gap junctions and by extracellular ATP. Am. J. Physiol. Lung Cell. Mol. Physiol. 280:L221–L228. 10.1152/ajplung.2001.280.2.L221 11159000

[bib34] Kao, J.P., J.M. Alderton, R.Y. Tsien, and R.A. Steinhardt. 1990. Active involvement of Ca^2+^ in mitotic progression of Swiss 3T3 fibroblasts. J. Cell Biol. 111:183–196. 10.1083/jcb.111.1.183 2114410 PMC2116168

[bib35] Kim, A.A., A. Nguyen, M. Marchetti, X. Du, D.J. Montell, B.L. Pruitt, and L.E. O’Brien. 2022. Independently paced Ca^2+^ oscillations in progenitor and differentiated cells in an ex vivo epithelial organ. J. Cell Sci. 135.14:jcs260249. 10.1242/jcs.260249 35722729 PMC9450890

[bib36] Koizumi, S., K. Fujishita, K. Inoue, Y. Shigemoto-Mogami, M. Tsuda, and K. Inoue. 2004. Ca^2+^ waves in keratinocytes are transmitted to sensory neurons: The involvement of extracellular ATP and P2Y2 receptor activation. Biochem. J. 380:329–338. 10.1042/bj20031089 14967069 PMC1224173

[bib37] De Koninck, P., and H. Schulman. 1998. Sensitivity of CaM kinase II to the frequency of Ca^2+^ oscillations. Science. 279:227–230. 10.1126/science.279.5348.227 9422695

[bib38] Kretz, M., C. Euwens, S. Hombach, D. Eckardt, B. Teubner, O. Traub, K. Willecke, and T. Ott. 2003. Altered connexin expression and wound healing in the epidermis of connexin-deficient mice. J. Cell Sci. 116:3443–3452. 10.1242/jcs.00638 12840073

[bib39] Kumamoto, J., M. Goto, M. Nagayama, and M. Denda. 2017. Real-time imaging of human epidermal calcium dynamics in response to point laser stimulation. J. Dermatol. Sci. 86:13–20. 10.1016/j.jdermsci.2017.01.002 28119009

[bib40] Laird, D.W. 2010. The gap junction proteome and its relationship to disease. Trends Cell Biol. 20:92–101. 10.1016/j.tcb.2009.11.001 19944606

[bib41] Liang, J., S. Balachandra, S. Ngo, and L.E. O’Brien. 2017. Feedback regulation of steady-state epithelial turnover and organ size. Nature. 548:588–591. 10.1038/nature23678 28847000 PMC5742542

[bib42] Liao, Y., K.H. Day, D.N. Damon, and B.R. Duling. 2001. Endothelial cell-specific knockout of connexin 43 causes hypotension and bradycardia in mice. Proc. Natl. Acad. Sci. USA. 98:9989–9994. 10.1073/pnas.171305298 11481448 PMC55565

[bib43] Madisen, L., A.R. Garner, D. Shimaoka, A.S. Chuong, N.C. Klapoetke, L. Li, A. van der Bourg, Y. Niino, L. Egolf, C. Monetti, . 2015. Transgenic mice for intersectional targeting of neural sensors and effectors with high specificity and performance. Neuron. 85:942–958. 10.1016/j.neuron.2015.02.022 25741722 PMC4365051

[bib44] Madisen, L., T. Mao, H. Koch, J.M. Zhuo, A. Berenyi, S. Fujisawa, Y.W.A. Hsu, A.J. Garcia III, X. Gu, S. Zanella, . 2012. A toolbox of Cre-dependent optogenetic transgenic mice for light-induced activation and silencing. Nat. Neurosci. 15:793–802. 10.1038/nn.3078 22446880 PMC3337962

[bib45] Marsh, E., D.G. Gonzalez, E.A. Lathrop, J. Boucher, and V. Greco. 2018. Positional stability and membrane occupancy define skin fibroblast homeostasis in vivo. Cell. 175:1620–1633.e13. 10.1016/j.cell.2018.10.013 30415836 PMC7605015

[bib46] Martin, P.E., and M. van Steensel. 2015. Connexins and skin disease: Insights into the role of beta connexins in skin homeostasis. Cell Tissue Res. 360:645–658. 10.1007/s00441-014-2094-3 25616557

[bib47] Matsubayashi, Y., M. Ebisuya, S. Honjoh, and E. Nishida. 2004. ERK activation propagates in epithelial cell sheets and regulates their migration during wound healing. Curr. Biol. 14:731–735. 10.1016/j.cub.2004.03.060 15084290

[bib85] Matsui, T., N. Kadono-Maekubo, Y. Suzuki, Y. Furuichi, K. Shiraga, H. Sasaki, A. Ishida, S. Takahashi, T. Okada, K. Toyooka, . 2021. A unique mode of keratinocyte death requires intracellular acidification. Proc Natl Acad Sci USA. 118:2020722118. 10.1073/pnas.2020722118 PMC8092583 33893234

[bib48] Mesa, K.R., P. Rompolas, G. Zito, P. Myung, T.Y. Sun, S. Brown, D.G. Gonzalez, K.B. Blagoev, A.M. Haberman, and V. Greco. 2015. Niche-induced cell death and epithelial phagocytosis regulate hair follicle stem cell pool. Nature. 522:94–97. 10.1038/nature14306 25849774 PMC4457634

[bib49] Mesa, K.R., K. Kawaguchi, K. Cockburn, D. Gonzalez, J. Boucher, T. Xin, A.M. Klein, and V. Greco. 2018. Homeostatic epidermal stem cell self-renewal is driven by local differentiation. Cell Stem Cell. 23:677–686.e4. 10.1016/j.stem.2018.09.005 30269903 PMC6214709

[bib50] Miura, K. 2020. Bleach correction ImageJ plugin for compensating the photobleaching of time-lapse sequences. F1000 Res. 9:1494. 10.12688/f1000research.27171.1 PMC7871415 33633845

[bib51] Mohammed, J., L.K. Beura, A. Bobr, B. Astry, B. Chicoine, S.W. Kashem, N.E. Welty, B.Z. Igyártó, S. Wijeyesinghe, E.A. Thompson, . 2016. Stromal cells control the epithelial residence of DCs and memory T cells by regulated activation of TGF-β. Nat. Immunol. 17:414–421. 10.1038/ni.3396 26901152 PMC5135085

[bib52] Moon, K.R., D. van Dijk, Z. Wang, S. Gigante, D.B. Burkhardt, W.S. Chen, K. Yim, A.V.D. Elzen, M.J. Hirn, R.R. Coifman, . 2019. Visualizing structure and transitions in high-dimensional biological data. Nat. Biotechnol. 37:1482–1492. 10.1038/s41587-019-0336-3 31796933 PMC7073148

[bib53] Murata, T., T. Honda, G. Egawa, Y. Yamamoto, R. Ichijo, F. Toyoshima, T. Dainichi, and K. Kabashima. 2018. Transient elevation of cytoplasmic calcium ion concentration at a single cell level precedes morphological changes of epidermal keratinocytes during cornification. Sci. Rep. 8:6610. 10.1038/s41598-018-24899-7 29700333 PMC5919969

[bib54] Muroyama, A., and T. Lechler. 2017. A transgenic toolkit for visualizing and perturbing microtubules reveals unexpected functions in the epidermis. eLife. 6:e29834. 10.7554/eLife.29834 28869035 PMC5605193

[bib55] Muzumdar, M.D., B. Tasic, K. Miyamichi, L. Li, and L. Luo. 2007. A global double-fluorescent Cre reporter mouse. Genesis. 45:593–605. 10.1002/dvg.20335 17868096

[bib56] Ohno, Y., and J.M. Otaki. 2015. Spontaneous long-range calcium waves in developing butterfly wings. BMC Dev. Biol. 15:17. 10.1186/s12861-015-0067-8 25888365 PMC4445562

[bib57] Park, S., C. Matte-Martone, D.G. Gonzalez, E.A. Lathrop, D.P. May, C.M. Pineda, J.L. Moore, J.D. Boucher, E. Marsh, A. Schmitter-Sánchez, . 2021. Skin-resident immune cells actively coordinate their distribution with epidermal cells during homeostasis. Nat. Cell Biol. 23:476–484. 10.1038/s41556-021-00670-5 33958758 PMC8603572

[bib58] Pineda, C.M., S. Park, K.R. Mesa, M. Wolfel, D.G. Gonzalez, A.M. Haberman, P. Rompolas, and V. Greco. 2015. Intravital imaging of hair follicle regeneration in the mouse. Nat. Protoc. 10:1116–1130. 10.1038/nprot.2015.070 26110716 PMC4632978

[bib59] Pnevmatikakis, E.A., and A. Giovannucci. 2017. NoRMCorre: An online algorithm for piecewise rigid motion correction of calcium imaging data. J. Neurosci. Methods. 291:83–94. 10.1016/j.jneumeth.2017.07.031 28782629

[bib60] Poenie, M., J. Alderton, R.Y. Tsien, and R.A. Steinhardt. 1985. Changes of free calcium levels with stages of the cell division cycle. Nature. 315:147–149. 10.1038/315147a0 3838803

[bib61] Pruitt, S.C., A. Freeland, M.E. Rusiniak, D. Kunnev, and G.K. Cady. 2013. Cdkn1b overexpression in adult mice alters the balance between genome and tissue ageing. Nat. Commun. 4:2626. 10.1038/ncomms3626 24149709 PMC3825507

[bib62] Ratan, R.R., F.R. Maxfield, and M.L. Shelanski. 1988. Long-lasting and rapid calcium changes during mitosis. J. Cell Biol. 107:993–999. 10.1083/jcb.107.3.993 3417787 PMC2115302

[bib63] Restrepo, S., and K. Basler. 2016. *Drosophila* wing imaginal discs respond to mechanical injury via slow InsP3R-mediated intercellular calcium waves. Nat. Commun. 7:12450. 10.1038/ncomms12450 27503836 PMC4980486

[bib64] Rieck, B., F. Sadlo, and H. Leitte. 2020. Topological machine learning with persistence indicator functions. In Topological Methods in Data Analysis and Visualization. Fifth edition. H. Carr, I. Fujishiro, F. Sadlo, and S. Takahashi, editors. Springer, Cham, Switzerland. 87–101. 10.1007/978-3-030-43036-8_6

[bib65] Rieder, C.L., and R.E. Palazzo. 1992. Colcemid and the mitotic cycle. J. Cell Sci. 102:387–392. 10.1242/jcs.102.3.387 1506421

[bib66] Romano, S.A., V. Pérez-Schuster, A. Jouary, J. Boulanger-Weill, A. Candeo, T. Pietri, and G. Sumbre. 2017. An integrated calcium imaging processing toolbox for the analysis of neuronal population dynamics. PLOS Comput. Biol. 13:e1005526. 10.1371/journal.pcbi.1005526 28591182 PMC5479595

[bib67] Rompolas, P., K.R. Mesa, K. Kawaguchi, S. Park, D. Gonzalez, S. Brown, J. Boucher, A.M. Klein, and V. Greco. 2016. Spatiotemporal coordination of stem cell commitment during epidermal homeostasis. Science. 352:1471–1474. 10.1126/science.aaf7012 27229141 PMC4958018

[bib68] Rosendo-Pineda, M.J., C.M. Moreno, and L. Vaca. 2020. Role of ion channels during cell division. Cell Calcium. 91:102258. 10.1016/j.ceca.2020.102258 32736154

[bib69] Sandryhaila, A., and J.M.F. Moura. 2013. Discrete signal processing on graphs: Graph fourier transform. 2013 IEEE Int. Conf. Acoust. Speech Signal Process.:6167–6170. 10.1109/ICASSP.2013.6638850

[bib70] Schweizer, J., M. Kinjo, G. Fürstenberger, and H. Winter. 1984. Sequential expression of mRNA-encoded keratin sets in neonatal mouse epidermis: Basal cells with properties of terminally differentiating cells. Cell. 37:159–170. 10.1016/0092-8674(84)90311-8 6202418

[bib71] Sender, R., and R. Milo. 2021. The distribution of cellular turnover in the human body. Nat. Med. 27:45–48. 10.1038/s41591-020-01182-9 33432173

[bib72] Shuman, D., S. Narang, P. Frossard, A. Ortega, and P. Vandergheynst. 2013. The emerging field of signal processing on graphs: Extending high-dimensional data analysis to networks and other irregular domains. IEEE Signal Process. Mag. 30:83–98. 10.1109/MSP.2012.2235192

[bib73] Snoeck, H.-W. 2020. Calcium regulation of stem cells. EMBO Rep. 21:e50028. 10.15252/embr.202050028 32419314 PMC7271657

[bib84] Stringer, C., M. Pachitariu, C.B. Reddy, M. Carandini, and K.D. Harris. 2018. Recordings of ten thousand neurons in visual cortex during spontaneous behaviors. Janelia Research Campus. FigShare. https://doi.org/10.25378/janelia.6163622.v6

[bib75] Stringer, C., M. Pachitariu, N. Steinmetz, C.B. Reddy, M. Carandini, and K.D. Harris. 2019. Spontaneous behaviors drive multidimensional, brainwide activity. Science. 364:255. 10.1126/science.aav7893 31000656 PMC6525101

[bib76] Tirosh, I., B. Izar, S.M. Prakadan, M.H. Wadsworth II, D. Treacy, J.J. Trombetta, A. Rotem, C. Rodman, C. Lian, G. Murphy, . 2016. Dissecting the multicellular ecosystem of metastatic melanoma by single-cell RNA-seq. Science. 352:189–196. 10.1126/science.aad0501 27124452 PMC4944528

[bib77] Tomasz, M. 1995. Mitomycin C: Small, fast and deadly (but very selective). Chem. Biol. 2:575–579. 10.1016/1074-5521(95)90120-5 9383461

[bib78] Tsutsumi, M., K. Inoue, S. Denda, K. Ikeyama, M. Goto, and M. Denda. 2009. Mechanical-stimulation-evoked calcium waves in proliferating and differentiated human keratinocytes. Cell Tissue Res. 338:99–106. 10.1007/s00441-009-0848-0 19657674

[bib79] Vasioukhin, V., L. Degenstein, B. Wise, and E. Fuchs. 1999. The magical touch: Genome targeting in epidermal stem cells induced by tamoxifen application to mouse skin. Proc. Natl. Acad. Sci. USA. 96:8551–8556. 10.1073/pnas.96.15.8551 10411913 PMC17554

[bib80] Wolf, F.A., P. Angerer, and F.J. Theis. 2018. SCANPY: Large-scale single-cell gene expression data analysis. Genome Biol. 19:15. 10.1186/s13059-017-1382-0 29409532 PMC5802054

[bib81] Xie, W., L.T. Chow, A.J. Paterson, E. Chin, and J.E. Kudlow. 1999. Conditional expression of the ErbB2 oncogene elicits reversible hyperplasia in stratified epithelia and up-regulation of TGFα expression in transgenic mice. Oncogene. 18:3593–3607. 10.1038/sj.onc.1202673 10380881

[bib82] Yuspa, S.H., A.E. Kilkenny, P.M. Steinert, and D.R. Roop. 1989. Expression of murine epidermal differentiation markers is tightly regulated by restricted extracellular calcium concentrations in vitro. J. Cell Biol. 109:1207–1217. 10.1083/jcb.109.3.1207 2475508 PMC2115750

