# Correction: Cell cycle controls long-range calcium signaling in the regenerating epidermis

**DOI:** 10.1083/jcb.20230209503052024c

**Published:** 2024-03-13

**Authors:** Jessica L. Moore, Dhananjay Bhaskar, Feng Gao, Catherine Matte-Martone, Shuangshuang Du, Elizabeth Lathrop, Smirthy Ganesan, Lin Shao, Rachael Norris, Nil Campamà Sanz, Karl Annusver, Maria Kasper, Andy Cox, Caroline Hendry, Bastian Rieck, Smita Krishnaswamy, Valentina Greco

Vol. 222, No. 7 | https://doi.org/10.1083/jcb.202302095 | April 27, 2023

After publication, the authors noticed that they had neglected to cite related papers by Ho and colleagues (Ho et al., 2021), Carlsson (Carlsson, 2009), and Cohen-Steiner and colleagues (Cohen-Steiner et al., 2007). In addition, the authors identified two errors in the Materials and methods section describing GSTH.

The following articles are now addressed in the text, as described above:

Carlsson, G. 2009. Topology and data. *Bull. Am. Math. Soc*. 46:255–308. https://doi.org/10.1090/S0273-0979-09-01249-X

Cohen-Steiner, D., H. Edelsbrunner, and J. Harer. 2007. Stability of persistence diagrams. *Discrete Comput. Geom*. 37:103–120. https://doi.org/10.1007/s00454-006-1276-5

Ho, K.Y.L., R.J. Khadilkar, R.L. Carr, and G. Tanentzapf. 2021. A gap-junction-mediated, calcium-signaling network controls blood progenitor fate decisions in hematopoiesis. *Curr. Biol*. 31:4697–4712. https://doi.org/10.1016/j.cub.2021.08.027

In the eighth paragraph of the Discussion section, the following sentence has been edited:

Therefore, our results build upon recent work (Ho et al., 2021) proposing a more nuanced role of gap junctions in mediating global communication across stem cell pools and suggest that Cx43 plays a unique role in coordinating information flow across this community of epidermal stem cells.

We have cited Gunnar Carlsson’s seminal work on TDA in the following sentence, which appears in the first paragraph of Step 4 in the Materials and methods section on GSTH: 

In persistent homology calculations, data is gradually coarse-grained by merging nearby points, and at each level of granularity, a graph (or simplicial complex in higher dimensions) of the data is quantified by counting the number of connected components, cycles, and potentially higher-dimensional “holes” (Carlsson, 2009).

In the same paragraph, the threshold ε was mis-printed as E, conflicting with the notation for trajectories E. The notation for filtration threshold has been corrected as shown below:

Here, we use a filtration method known as the Vietoris-Rips filtration, where we create connections between two points, e_i_ and e_j_, in the trajectory if the points are closer than a threshold ε, measured using the Euclidean distance on the embedding. The threshold ε is gradually increased, ranging from 0 to ∞, until all points are connected in a fully connected graph. 

We have also modified a sentence describing persistent homology in the second paragraph of Step 4 in the Materials and methods section on GSTH to mention stability properties of homological features:

Persistent homology is still under-explored in data science, but it is a robust tool for analyzing the shape features of datasets across multiple scales with guaranteed stability properties, i.e., ensuring small changes in the input data lead to small changes in the homological features (Cohen-Steiner et al., 2007).

Equation 5, which describes the Wasserstein distance, was previously incomplete. It has been corrected as shown below:


$$W_{2}\left(Q_{1},Q_{2}\right)=\underset{m:Q_{1}\to Q_{2}}{\inf}{\left(\sum_{q_{1}\in Q_{1}}{\left\|q_{1}-m\left(q_{1}\right)\right\|}^{m}\right)}^{\frac{1}{m}}$$

The errors appear in print and in PDFs downloaded on or before March 11, 2024. The authors apologize for the errors and any confusion.

